# Plasma protein biomarkers reflective of the host response in patients developing Intensive Care Unit-acquired pneumonia

**DOI:** 10.1186/s13054-023-04536-0

**Published:** 2023-07-06

**Authors:** Tjitske S. R. van Engelen, Tom D. Y. Reijnders, Fleur P. Paling, Marc J. M. Bonten, Leen Timbermont, Surbhi Malhotra-Kumar, Jan A. J. W. Kluytmans, Hessel Peters-Sengers, Tom van der Poll, for the ASPIRE-I. C. U. Study Team, Martin Wolkewitz, Omar Ali, Alexey Ruzin, Leen Timbermont, Christine Lammens, Sebastiaan Hullegie, Darren Troeman, Denise van Hout, Daniël Prins, Rubana Kalyani, Mark Eickhoff, Kathryn Shoemaker, Tuba Vilken, Jelle Vlaeminck, Jasmine Coppens, Thomas van der Schalk, Basil Britto Xavier, Evelina Odisseeva, Rossitza Vatcheva, Michal Drab, Jaromir Vajter, Kadri Tamme, Muriel Fartoukh, Alain LePape, Mickael Landais, Gaetan Plantefève, Evelina Tacconelli, Achim Kaasch, Róbert Jurkinya, Iványi Zsolt, Miranda van Rijen, Olaf Cremer, Biljana Carevic, Jasna Jevdjić, Dolores Escudero, Miguel Sanchez Garcia, Cristina Prat-Aymerich, Borja Suberviola-Cañas, Angel Arenzana-Seisdedos, Hürrem Bodur, Cenk Kirakli, Ilkay Bozkurt, Sandra Long

**Affiliations:** 1 Center for Experimental and Molecular Medicine (CEMM), Amsterdam University Medical Centers, Location AMC, University of Amsterdam, Room G2-105, Meibergdreef 9, 1105 AZ Amsterdam, The Netherlands; 2 Julius Center for Health Sciences and Primary Care, University Medical Center Utrecht, Utrecht University, Utrecht, The Netherlands; 3 Laboratory of Medical Microbiology, Vaccine and Infectious Disease Institute, University of Antwerp, Antwerp, Belgium; 4 Department of Medical Microbiology, University Medical Center Utrecht, Utrecht University, Utrecht, The Netherlands; 5 Division of Infectious Diseases, Amsterdam University Medical Centers, University of Amsterdam, Amsterdam, The Netherlands

**Keywords:** Critical care, Respiratory tract infections, Biomarkers

## Abstract

**Background:**

Immune suppression has been implicated in the occurrence of pneumonia in critically ill patients. We tested the hypothesis that Intensive Care Unit (ICU)-acquired pneumonia is associated with broad host immune aberrations in the trajectory to pneumonia, encompassing inflammatory, endothelial and coagulation responses. We compared plasma protein biomarkers reflecting the systemic host response in critically ill patients who acquire a new pneumonia (cases) with those who do not (controls).

**Methods:**

We performed a nested case–control study in patients undergoing mechanical ventilation at ICU admission with an expected stay of at least 48 h enrolled in 30 hospitals in 11 European countries. Nineteen host response biomarkers reflective of key pathophysiological domains were measured in plasma obtained on study inclusion and day 7, and—in cases—on the day of pneumonia diagnosis.

**Results:**

Of 1997 patients, 316 developed pneumonia (15.8%) and 1681 did not (84.2%). Plasma protein biomarker analyses, performed in cases and a randomly selected subgroup of controls (1:2 ratio to cases, *n* = 632), demonstrated considerable variation across time points and patient groups. Yet, cases showed biomarker concentrations suggestive of enhanced inflammation and a more disturbed endothelial barrier function, both at study enrollment (median 2 days after ICU admission) and in the path to pneumonia diagnosis (median 5 days after ICU admission). Baseline host response biomarker aberrations were most profound in patients who developed pneumonia either shortly (< 5 days, *n* = 105) or late (> 10 days, *n* = 68) after ICU admission.

**Conclusions:**

Critically ill patients who develop an ICU-acquired pneumonia, compared with those who do not, display alterations in plasma protein biomarker concentrations indicative of stronger proinflammatory, procoagulant and (injurious) endothelial cell responses.

*Trial registration*: ClinicalTrials.gov Identifier: NCT02413242, posted April 9th, 2015.

**Graphical abstract:**

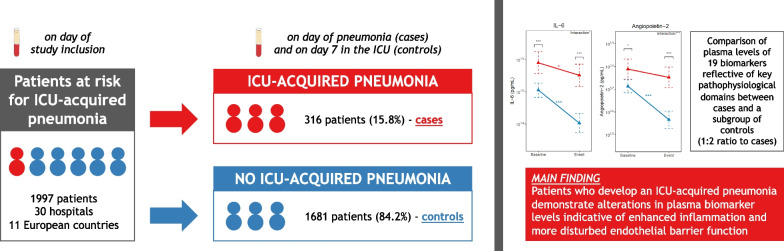

**Supplementary Information:**

The online version contains supplementary material available at 10.1186/s13054-023-04536-0.

## Background

Intensive Care Unit (ICU)-acquired pneumonia is one of the most frequently diagnosed infections in the ICU, with a—relative—attributable mortality of 13% [1, 2]. Important risk factors for ICU-acquired pneumonia include invasive procedures (particularly mechanical ventilation), the underlying medical condition, comorbidities, and severity of disease [3, 4]. In recent years much attention has been given to host response deviations in critically ill patients, including those with a sepsis admission diagnosis, that may render them vulnerable to secondary opportunistic infections [5–8]. In this context, the vast majority of research focused on critical illness-associated immune suppression as a key factor placing ICU patients at risk for infection [5–8]. Our group previously reported on host response aberrations in critically ill patients with sepsis prior to the development of ICU-acquired infections and found that these patients, rather than merely showing signs of immune suppression, demonstrated wide-ranging disturbances across multiple pathophysiological domains when compared with patients who did not develop an ICU-acquired infection [9]. Additional studies have provided evidence for a sustained and complex dysregulation of the host response entailing both immune suppression and hyperinflammation in patients who remain on the ICU for prolonged periods of time, who oftentimes develop a chronic critical illness termed “persistent inflammation, immunosuppression and catabolism syndrome” or PICS [7].

We here tested the hypothesis that critically ill patients, irrespective of their primary reason for admission, exhibit broad anomalies in their host response both prior to and during ICU-acquired pneumonia, and that these are distinctive from patients who do not acquire pneumonia while on the ICU. For this, we measured 19 host response biomarkers providing insight into key pathophysiological pathways in plasma samples collected in a prospective observational study in patients admitted to 30 ICUs throughout Europe with various admission diagnoses (medical, trauma, surgical) and compared patients who developed pneumonia during their ICU stay (cases) with patients who did not develop pneumonia (controls).

Our study aimed to obtain insight into: (1) host response protein differences between cases and controls prior to development of ICU-acquired pneumonia in the former group; (2) host response protein aberrations at the time of pneumonia diagnosis; (3) host response protein trajectories, i.e., the change in host response over time from prior to ICU-acquired pneumonia to the day of ICU-acquired pneumonia.

## Methods

### Patient population

This study was conducted as part of the “Advanced understanding of *Staphylococcus aureus* and *Pseudomonas aeruginosa* Infections in EuRopE—Intensive Care Units” (ASPIRE-ICU) project, a study of adult ICU patients at 30 hospitals in 11 European countries that recruited participants between June 2015 and October 2018 [10]. Study methods have been reported elsewhere [11]. Briefly, patients with an expected length of ICU stay of 48 h or more and who underwent mechanical ventilation at ICU admission (or were expected to undergo ventilation within 24 h) were enrolled within 3 days after ICU admission in a 1:1 ratio of *Staphylococcus *(*S.*) *aureus*-colonized (identified by screening on admission) and non-colonized patients. The study protocol was approved by the institutional review boards or ethical committees in each country and/or site, and all participants or their legally authorized representative provided written, informed consent for additional data and sample collection. The protocol definition of an ICU-acquired pneumonia was described in detail elsewhere [11] and in Additional file 1. Briefly, four clinical criteria were assessed daily: new antibiotic use, new blood cultures performed, new chest radiograph or computed tomography done, or another new reason to suspect pneumonia. In cases with one positive answer, a combination of major and minor criteria was assessed to categorize patients as having protocol-defined pneumonia, or not [11]. The diagnosis of pneumonia, based on criteria assessed daily, triggered the collection of an “event” blood sample; there were no cases in whom the diagnosis pneumonia was later refuted.

The current project was designed as a nested case–control study within the ASPIRE-ICU population. A case was defined as a patient who developed a (protocol-defined) ICU-acquired pneumonia at least 48 h after ICU admission. A control was defined as a patient who did not develop a protocol-defined ICU-acquired pneumonia. Comorbidities and causative pathogens were defined as described in Additional file 1.

### Sample collection and assays

Ethylenediaminetetraacetic acid (EDTA) anticoagulated blood was obtained upon enrollment into ASPIRE-ICU (baseline). In cases, a follow-up blood sample was obtained on the day pneumonia was diagnosed and on day 7 after inclusion (Additional file 1: Fig. S1); in controls, a follow-up sample was drawn on day 7 after inclusion. Biomarkers were categorized into four pathophysiological domains: interleukin (IL)-6, IL-8, IL-10, and IL-1 receptor antagonist (IL-1RA)(reflecting cytokine release); matrix metalloproteinase (MMP)-8, soluble triggering receptor expressed on myeloid cells (sTREM)-1, soluble cluster of differentiation (sCD)163, soluble receptor for advanced glycation endproducts (sRAGE), tenascin-C and procalcitonin (reflecting systemic inflammation); sE-selectin, soluble vascular cell adhesion protein (sVCAM)-1, fractalkine, syndecan-1, soluble thrombomodulin, angiopoietin-1, and angiopoietin-2 (reflecting endothelial activation and function); soluble tissue factor and D-dimer (reflecting coagulation activation). For further details, see Additional file 1.

### Statistical analysis

All biomarker values were logarithmically transformed and analyzed using linear mixed model analyses. Calculation of principal component analysis (PCA) plots was done by a singular value decomposition of the centered and scaled data matrix including the (logged) protein plasma biomarkers for key pathophysiological pathways. The mixed model was fitted taking the group (cases versus controls), the discrete timepoint (i.e., baseline, event—only for cases—and/or day 7), and their interaction as fixed effects, and patient-specific intercepts as random effects unless otherwise stated. The interaction term (group × timepoints) revealed whether there was a difference in biomarker trajectory over time between cases and controls, where the patient-specific intercept accounted for repeated measures within each patient. In additional analyses, we adjusted for the following predefined variables that may confound the relationship between ICU-acquired pneumonia and plasma biomarker levels: site of enrollment, age, sex, body mass index, Charlson comorbidity index, reason for admission (medical, surgery, trauma), *S. aureus* colonization status, Acute Physiology And Chronic Health Evaluation (APACHE)-IV score, and immunosuppressed status. Two additional analyses were performed: (1) biomarker trajectory in the subgroup of cases in whom also a sample was taken after the event of ICU-acquired pneumonia, and (2) the association between the time to develop an ICU-acquired pneumonia and baseline biomarker levels. For more details, see the statistical paragraph of Additional file 1.

## Results

### Patient characteristics and clinical outcomes

**Fig. 1:**
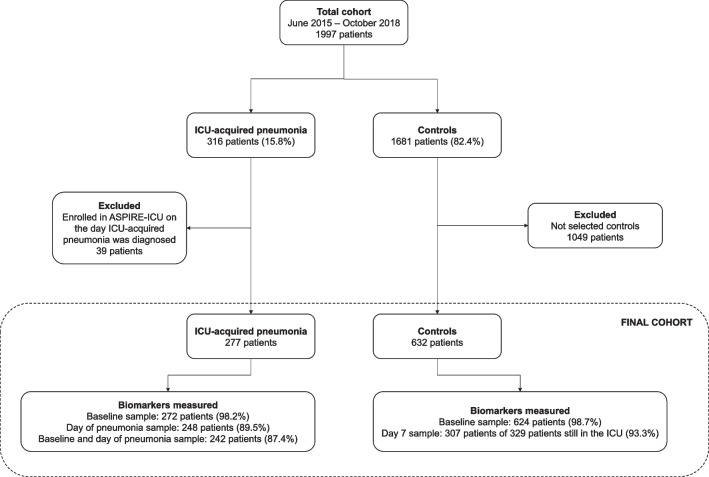
Flowchart of patient inclusion. *ICU* Intensive Care Unit

ASPIRE-ICU enrolled 1997 patients, of whom 316 (15.8%) developed ICU-acquired pneumonia (cases) and 1681 (84.2%) did not (controls) (Fig. 1). From all controls, we randomly selected a subset in a 2:1 ratio to the cases, resulting in 632 controls for host response biomarker analyses. Selected (*n* = 632) and not selected (*n* = 1049) controls did not differ regarding baseline characteristics and clinical outcomes (Additional file 1: Table S1). Table 1 shows baseline characteristics at ICU admission and clinical outcomes of the study population; missing clinical data is depicted in Additional file 1: Table S2. At baseline, cases were similar to controls, with the exception that cases more often had immunosuppression as comorbidity (7.9% versus 4.4% in controls, *P* = 0.04) and less often mild liver disease (0.3% versus 3.5%, *P* = 0.01; Additional file 1: Table S3). In cases the median interval from ICU admission to the diagnosis of ICU-acquired pneumonia was 5 days (interquartile range [IQR] 3–9 days). The causative pathogen was *S. aureus* in 140 patients (44.3%) and *Pseudomonas *(*P.*) *aeruginosa* in 48 patients (15.2%); in 21 patients (6.6%) both pathogens were assigned as causative. Cases had a longer length of ICU stay, higher readmission rates, and a higher 90-day mortality.

**Table 1:** Patient characteristics and clinical outcome

	Cases (*n* = 316)	Controls (*n* = 632)	*P* value
*Baseline data*			
Age, year, mean (SD)	64 (15.3)	63 (16.0)	0.34
Female sex, *n* (%)	96 (30.4)	227 (35.9)	0.11
Body Mass Index, mean (SD)	27.3 (6.1)	27.2 (6.0)	0.97
Charlson comorbidity index, median (IQR)	3 (2–5)	3 (2–5)	0.98
Origin prior to ICU admission, *n* (%)			0.56
Home	173 (54.7)	326 (51.6)	
General ward	86 (27.2)	178 (28.2)	
Long term facility	7 (2.2)	14 (2.2)	
Other ICU	39 (12.3)	97 (15.3)	
Primary reason for ICU admission, *n* (%)			0.07
Medical	166 (52.5)	292 (46.2)	
Surgical, cardiothoracic	11 (3.5)	44 (7.0)	
Surgical, other	75 (23.7)	171 (27.1)	
Trauma	64 (20.3)	125 (19.8)	
Colonized with *Staphylococcus aureus* on admission, n (%)	172 (54.4)	316 (50.0)	0.22
Pneumonia on ICU admission, n (%)	75 (23.7)	124 (19.6)	0.17
APACHE IV, mean (SD)	75 (37.1)	70 (38.0)	0.07
Laboratory values at ICU admission^a^			
White blood cells, 10^9^/L, median (IQR)	12.6 (8.6–18.1)	13.0 (9.2–17.5)	0.54
Neutrophils, 10^9^/L, median (IQR)	10.8 (7.1–15.1)	11.0 (7.2–15.2)	0.85
Monocytes, 10^9^/L, median (IQR)	0.7 (0.4–1.0)	0.7 (0.4–1.1)	0.74
Lymphocytes, 10^9^/L, median (IQR)	0.8 (0.5–1.2)	0.8 (0.5–1.3)	0.97
Platelets, 10^9^/L, median (IQR)	199 (139–266)	205 (148–271)	0.34
*Outcome data*			
Length of ICU stay, days, median (IQR)	15 (10–28)	8 (5–14)	< 0.001
Readmission < 30 days of ICU discharge, n (%)	16 (5.1)	21 (3.3)	< 0.001
Status at Day 90 after ICU admission, n (%)			0.02
Alive	158 (53.6)	368 (63.9)	
Dead	137 (46.4)	208 (36.1)	

*APACHE IV*—Acute Physiology and Chronic Health Evaluation IV; *ICU*—Intensive Care Unit; *IQR*—interquartile range. A case was defined as a subject who developed a (by protocol defined) ICU-acquired pneumonia. A control was defined as a subject who did not develop a protocol-defined ICU-acquired pneumonia. From all controls (n = 1681) we randomly selected a subset in a 2:1 ratio to cases. Continuous nonparametric data were analyzed using a Wilcoxon signed rank sum- or Kruskal–Wallis test; categorical data were analyzed using a Fisher exact test; continuous parametric data were analyzed using a Student t test; a *P* value < 0.05 was considered statistically significant

^a^Neutrophil, monocyte, and lymphocyte counts missing in up to 47% of patients (considered missing at random), see Additional file 1: Table S2 for details

### Baseline host response protein differences between patients who did and those who did not develop an ICU-acquired pneumonia

**Fig. 2:**
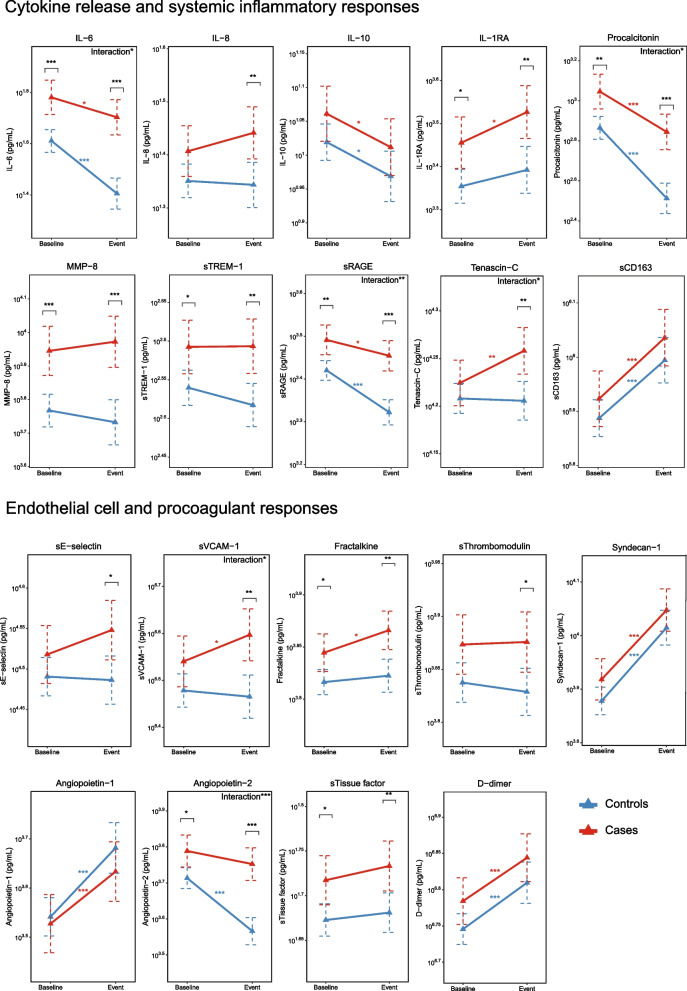
Plasma protein biomarkers indicative of cytokine release and systemic inflammatory responses, and endothelial cell and procoagulant responses, in Intensive Care Unit (ICU) patients stratified according to the development of an ICU-acquired pneumonia (case) or not (control) at baseline, event, and their change over time. The baseline sample was obtained upon enrollment into the study. In cases, a follow-up blood sample was obtained on the day the pneumonia was diagnosed (event); in controls, a follow-up sample (”event”) was drawn on day 7 after enrollment into the study. Data are expressed as mean estimate with 95 percent confidence interval, derived from the linear mixed model. Asterisks indicate differences between groups (*P < 0.05. **P < 0.01. ***P < 0.001). *Definition of abbreviations:* *CD*—cluster of differentiation, *IL*—interleukin, *MMP*—matrix metalloproteinase, *RA*—receptor antagonist, *RAGE*—receptor for advanced glycation endproducts, *s*—soluble, *TREM*—triggering receptor expressed on myeloid cells; *VCAM*—vascular cell adhesion protein

Our first study objective was to obtain insight into host response protein differences between cases and controls prior to development of ICU-acquired pneumonia in the former group. 39 cases (12.3%) were enrolled in ASPIRE-ICU on the day ICU-acquired pneumonia was diagnosed; these patients were excluded from the biomarker analysis since a blood sample prior to development of pneumonia was lacking (Fig. 1). Of the remaining 277 cases, 272 baseline blood samples (98.2%) were obtained 2 [1, 2] days (median [IQR]) after ICU admission, and 4 [2–8] days prior to the diagnosis of ICU-acquired pneumonia (Additional file 1: Fig. S1). From 632 controls 624 baseline blood samples (98.7%) were available, drawn 2 [1, 2] days after ICU admission. We analyzed 19 biomarkers reflective of four pathophysiological pathways implicated in host response aberrations during severe infection: cytokine release and systemic inflammation, and endothelial cell and procoagulant responses. On all four domains cases and controls already differed at baseline (Additional file 1: Fig. S2). Baseline plasma levels of the cytokines IL-6 and IL-1RA were higher in cases than controls, while the plasma concentrations of IL-8 and the anti-inflammatory cytokine IL-10 were not different between groups. Likewise, systemic inflammation markers procalcitonin, MMP-8, sTREM-1, and sRAGE were higher at baseline in cases, while tenascin-C and sCD163 concentrations were not different between groups (Fig. 2; for direct comparison see Additional file 1: Fig. S3). Cases also displayed higher levels of the endothelial cell markers fractalkine and angiopoietin-2 (indicative of disturbed endothelial barrier function), while other endothelial cell markers (sE-selectin, sVCAM-1, sThrombomodulin, syndecan-1, angiopoietin-1) were similar between groups. Regarding coagulation markers, baseline sTissue factor levels were higher in cases; D-dimer was similar between groups (Fig. 2; for direct comparison see Additional file 1: Fig. S4). Similar results were obtained after adjustment for potential confounders (Additional file 1: Table S4). Collectively, these data show that patients who later during their ICU stay develop pneumonia have more exaggerated host response protein aberrations at baseline than those who do not acquire pneumonia.

### Host response proteins at the time of ICU-acquired pneumonia diagnosis relative to controls

We next compared host response proteins in cases at the time of the diagnosis of ICU-acquired pneumonia with those in controls at day 7 after inclusion. Of 277 cases, 248 event samples (89.5%) were obtained at the day of ICU-acquired pneumonia diagnosis (at a median of 6 days [IQR, 4–10] after ICU admission. Of 632 controls, 307 follow-up samples (48.6% of all selected controls, 93.3% of the 329 controls still in the ICU) were obtained on day 7 after inclusion (8 [7, 8] days after ICU admission) and available (Fig. 1); of 303 controls who were not in the ICU anymore at day 7, 234 had been discharged and 68 had died (no data available for one patient). Analyzing the same four pathophysiological domains at the time of the event, differences were more profound in cases compared to controls than at baseline (Additional file 1: Fig. S2). Cytokine levels (IL-6, IL-8, IL-1RA) were higher in cases than controls; IL-10 levels were not different between groups; regarding systemic inflammation procalcitonin, MMP-8, sTREM-1, sRAGE and tenascin-C were higher in cases, while sCD163 was not different between groups (Fig. 2; Additional file 1: Fig. S3). In the endothelial domain, cases had higher levels of sE-selectin, sVCAM-1, fractalkine, sThrombomodulin and angiopoietin-2; other endothelial cell markers (syndecan-1, angiopoietin-1) were similar between groups (Fig. 2; Additional file 1: Fig. S4). The levels of the coagulation marker sTissue factor but not D-dimer were higher in cases than controls (Fig. 2; Additional file 1: Fig. S4). Similar results were obtained after adjustment for potential confounders (Additional file 1: Table S4). Together these results suggest that the presence of ICU-acquired pneumonia is associated with stronger host response protein aberrations as compared to critical illness in the absence of ICU-acquired pneumonia.

### Host response protein trajectories

We next sought to obtain insight into host response protein trajectories from baseline to the diagnosis of ICU-acquired pneumonia, and differences with host response protein trajectories in controls remaining in the ICU without acquiring pneumonia. With regard to cytokine release and systemic inflammation IL-1RA, tenascin-C, and sCD163 increased from baseline to the day of pneumonia diagnosis, IL-6, IL-10, procalcitonin and sRAGE decreased, and IL-8, MMP-8, and sTREM-1 remained unchanged in cases (Fig. 2). With regard to endothelial cell and procoagulant responses, sVCAM-1, fractalkine, syndecan-1, angiopoietin-1 and D-dimer increased from baseline to event, and sE-selectin, sThrombomodulin, angiopoietin-2, and sTissue factor remained unchanged in cases (Fig. 2). In controls, cytokine release and systemic inflammation responses decreased from baseline until day 7 (IL-6, IL-10, procalcitonin, and sRAGE), while sCD163 increased over time. Endothelial cell and procoagulant responses in controls increased (syndecan-1, angiopoietin-1, and D-dimer), and angiopoietin-2 decreased. Importantly, directly comparing biomarker trajectories between cases and controls revealed several differences: the levels of IL-6, procalcitonin, sRAGE, and angiopoietin-2 decreased less strongly over time in cases, while levels of tenascin-C and sVCAM-1 increased in cases but remained stable in controls (Fig. 2; Additional file 1: Table S4). These differences in trajectories remained significant after adjusting for potential confounders at baseline (Additional file 1: Table S4). Collectively, these data show that host response protein biomarkers of patients who develop ICU-acquired pneumonia, relative to patients who do not develop ICU-acquired pneumonia, show more strongly altered plasma levels across distinct pathophysiological domains in a sustained way, i.e., from briefly after ICU admission up to the day of the diagnosis of pneumonia.

In 122 cases (44% of all cases) the day of the pneumonia occurred prior to day 7 after inclusion (a standard sampling day; Additional file 1: Table S5). In this subgroup we conducted paired analyses across three time points, providing insight in biomarker trajectories after pneumonia diagnosis. The levels of IL-6, procalcitonin, sRAGE, and angiopoietin-2 were lower post-event compared to the day of pneumonia diagnosis, while syndecan-1 and angiopoietin-1 concentrations were higher post-event; the other host response biomarkers remained unaltered (Additional file 1: Figs. S5 and S6). These results suggest that, while most responses persist, some inflammatory markers decline and some endothelial markers continue to rise following pneumonia treatment, reflecting the heterogeneity of biomarker trajectories in critically ill patients.

Lastly, we explored if host response protein aberrations detected in cases at baseline were influenced by the time interval between admission and the occurrence of pneumonia (Additional file 1: Table S6). We hypothesized that in patients who developed pneumonia shortly after inclusion might show baseline biomarker differences (relative to controls) resulting from an emerging infection of the airways that was not yet detected. For most host response biomarkers we observed nonlinear trends in their trajectories as such that patients who developed ICU-acquired pneumonia either early after admission (2–5 days) or very late (> 10 days) showed stronger host response protein changes at baseline as compared to patients who developed pneumonia between 6 and 10 days (Additional file 1: Fig. S7 and S8).

## Discussion

Pneumonia is one of the most common nosocomial infections in the ICU and there is evidence that immune suppression resulting from critical illness puts patients at risk for secondary infections [5–8]. We here examined the possibility that critically ill patients exhibit broad disturbances in their host response across several pathophysiological domains both prior to and during ICU-acquired pneumonia by sequentially measuring 19 protein biomarkers in plasma. We show that plasma biomarker levels, while heterogeneous across time points and patient groups, were different in patients who later during their ICU stay develop pneumonia from to those in patients who do not acquire pneumonia, already shortly after admission as well as in the trajectory toward the day pneumonia is diagnosed.

The majority of studies published on the association between immune changes and the development of secondary infections in the ICU focused on immune suppressive features of especially mononuclear cells, such as their reduced responsiveness to bacterial components, impaired antigen presentation capacity and signs of apoptosis [5–8]. Reduced HLA-DR expression on circulating monocytes and lymphocytopenia, both considered features of immune suppression, have been associated with an increased risk on nosocomial infections following sepsis or trauma [12–15]. Additionally, patients with ventilator-associated pneumonia showed lower CD4+ T cell counts and a reduced capacity of monocytes to release proinflammatory cytokines upon ex vivo stimulation, as compared with patients with non-respiratory nosocomial infections [16], and blood leukocyte gene expression profiles also suggested immune suppression in patients with ICU-acquired pneumonia [17]. Notably, while critical illness without doubt is associated with various immune suppressive reactions [5–8], patients admitted to the ICU concurrently show systemic hyperinflammatory responses which include cytokine release, and activation of the coagulation system and the vascular endothelium [6, 9, 18]. Our group recently reported that in ICU patients the degree of reduction in cytokine release by blood leukocytes, a common readout of immune suppression, was associated with simultaneously increasing systemic inflammation, stronger endothelial cell activation, loss of endothelial barrier integrity and enhanced procoagulant responses, suggesting that the strongest immune suppression occurs in those patients who concurrently display signs of stronger systemic inflammation [19]. In agreement, another study reported an inverse relationship between the plasma levels of IL-6, MMP-8 and CXCL9 (indicative of systemic inflammation) and the TNF production capacity of whole blood obtained from patients with sepsis [20]. Moreover, patients admitted for sepsis who later developed a secondary infection while in the ICU demonstrated greater aberrations in plasma biomarkers reflective of these hyperinflammatory pathophysiological domains relative to patients who did not develop an ICU-acquired infection [9]. The present investigation further supports the concept that secondary infections are associated with a widely disturbed immune response, encompassing distinct mediator systems, and characterized by not only immune suppression but also hyperinflammation. This notion is further reinforced by a study in trauma patients in whom multiple proinflammatory mediators were elevated within the first 24 h after trauma in those who subsequently developed a nosocomial infection [21]. Likewise, in patients with sepsis elevated plasma midregional-proadrenomedulin levels were associated with an increased frequency of secondary infections [22]. Of note, differences in plasma biomarkers between cases and controls occurred despite similar disease severities at ICU admission.

Changes in plasma host response proteins at study enrollment were most profound in patients in whom pneumonia was diagnosed relatively shortly (< 5 days) or late (> 10 days) after ICU admission. While the time windows chosen are arbitrarily, possibly these groups represent distinct pathobiological phenotypes, with in the first group changes that were partially already the consequence of an evolving infectious process in the lower airways, and in the latter group alterations with long-term impact on the susceptibility to pneumonia. To our knowledge such time-dependent analyses in the context of ICU-acquired infections have not been performed previously. Additional studies, with prospective sequential sampling, are warranted to obtain further insight into the immunopathobiology preceding secondary infections in critically ill patients.

The current study is different from and expands our earlier investigation in which we reported on ICU-acquired infections in patients with sepsis in several ways [9]. The present study includes a more heterogeneous population of critically ill patients with various admission reasons (rather than only sepsis) and focusses on ICU-acquired pneumonia specifically (rather than all ICU-acquired infections combined). We determined biomarker trajectories to the day pneumonia was diagnosed, and in controls to day 7 after enrollment (rather than measurements restricted during the first 4 days after ICU admission). Furthermore, this study enrolled patients from 30 hospitals, both academic and non-academic, across Europe (rather than from two academic hospitals in the Netherlands). The plasma biomarker panels reported in both studies partially overlap (IL-6, IL-8, IL-10, MMP-8, fractalkine, sE-selectin, angiopoietin-1 and -2). As compared with our previous study, in the current analyses additional inflammatory markers (procalcitonin, sTREM-1, sRAGE, tenascin-C), more (specific) endothelial cell markers (sVCAM-1, syndecan-1, sThrombomodulin) and an additional coagulation marker (sTissue factor) were measured, while platelet counts, anticoagulant proteins (antithrombin, protein C) and prothrombin time were not measured.

Our study has strengths and limitations. Due to the inclusion criteria the study population was relatively enriched for patients with *S. aureus* colonization, which may hamper generalization of results. However, adjusting for *S. aureus* colonization status did not change results on host response differences between cases and controls. We provide information on a large, well-defined, prospectively collected cohort including patients admitted to ICUs throughout 30 hospitals in Europe, which enhances the generalizability of the results. Nonetheless, while representative of a general ICU population, study patients comprised a heterogeneous group with various underlying diseases that may impact biomarker responses. The disease of interest, ICU-acquired pneumonia, was extensively protocol-defined and assessed daily in all enrolled patients. However, the embedding of this large investigation across multiple study sites precluded functional and/or cell-specific measurements requiring fresh samples (e.g., blood leukocyte cytokine production capacity and monocyte HLA-DR expression), and systematic sampling of the airways. Only admission types were registered and specific diagnoses for “medical” admissions were not recorded. Analyses of the biomarker trajectories were limited by the fact that 303 controls (47.9%) were not in the ICU anymore at day 7 due to discharge or death, competing risks that may impact the results in opposite directions.

## Conclusions

Immune stimulatory therapy has been suggested as a novel approach to treat sepsis-induced immune suppression in order to reduce the occurrence of secondary infections and late mortality [5, 8]. We here report that critically ill patients developing ICU-acquired pneumonia show changes in plasma protein biomarkers that are indicative of a more broadly disturbed host response entailing several proinflammatory reactions prior to development of pneumonia as compared to critically ill patients who did not develop ICU-acquired pneumonia. Together, these data suggest that critically ill patients who develop pneumonia while on the ICU show heterogeneous baseline immune alterations and that the broad application of immune stimulatory therapy may be harmful in some patients. Additional observational studies should focus on identifying biological factors that may inform the personalized application of immunomodulatory (stimulation versus suppression) therapies in critically ill patients at risk of ICU-acquired infections.

### Supplementary Information

**Additional file 1**: STROBE statement; Supplementary methods: Definition of ICU-acquired pneumonia; Comorbidities; Causative pathogens; Sample collection; Assays; Statistical analysis; References; Supplementary tables: **Table S1**. Patient characteristics and clinical outcome of selected and not selected controls; **Table S2**. Missing clinical data; **Table S3**. Comorbidities; **Table S4**. Overview of linear mixed model estimates comparing cases with controls at baseline and onset of ICU-acquired pneumonia; **Table S5**. Patient characteristics and clinical outcome of cases in whom pneumonia occurred prior to day 7; **Table S6**. Patient characteristics of cases categorized into three groups according to the day of onset of ICU-acquired pneumonia; Supplementary figures: **Fig. S1**. Schematic representation of the clinical course of 277 cases during ICU-Stay; **Fig. S2**. Plasma protein biomarkers indicative of cytokine release and systemic inflammatory responses, and endothelial cell and procoagulant responses in Intensive Care Unitpatients stratified according to the development of an ICU-acquired pneumoniaor notat baseline and the time of the event. **Fig. S3**. Cytokine release and systemic inflammatory responses in ICU patients stratified according to the development of ICU-acquired pneumonia or not at baseline and event; **Fig. S4**. Endothelial cell and procoagulant responses in ICU patients stratified according to the development of ICU-acquired pneumonia or not at baseline and event; **Fig. S5**. Cytokine and systemic inflammatory responses in patients in whom pneumonia occurred prior to day 7; **Fig. S6**. Endothelial cell and procoagulant responses in Intensive Care Unitpatients in whom pneumonia occurred prior to day 7; **Fig. S7**. Baseline cytokine release and systemic inflammatory responses in ICU patients who developed ICU acquired pneumonia; **Fig. S8**. Baseline endothelial cell and procoagulant responses in ICU patients who developed ICU-acquired pneumonia; ASPIRE-ICU Study Team.

## Data Availability

The data and code that support the findings of this study are available from the corresponding author, TvE, upon reasonable request.

## Abbreviations

APACHE: Acute physiology and chronic health evaluation
CD: Cluster of differentiation
EDTA: Ethylenediaminetetraacetic acid
ICU: Intensive Care Unit
IL: Interleukin
IQR: Interquartile range
MMP: Matrix metalloproteinase
*P. aeruginosa*: *Pseudomonas aeruginosa*
RA: Receptor antagonist
RAGE: Receptor for advanced glycation endproducts
*s*: Soluble
*S. aureus*: *Staphylococcus aureus*
SD: Standard deviation
TREM: Triggering receptor expressed on myeloid cells
VCAM: Vascular cell adhesion protein
