# Correction: Plasma protein biomarkers reflective of the host response in patients developing Intensive Care Unit-acquired pneumonia

**DOI:** 10.1186/s13054-023-04700-6

**Published:** 2023-11-01

**Authors:** Tjitske S. R. van Engelen, Tom D. Y. Reijnders, Fleur P. Paling, Marc J. M. Bonten, Leen Timbermont, Surbhi Malhotra-Kumar, Jan A. J. W. Kluytmans, Hessel Peters-Sengers, Tom van der Poll, for the ASPIRE-I. C. U. Study Team, Martin Wolkewitz, Omar Ali, Alexey Ruzin, Leen Timbermont, Christine Lammens, Sebastiaan Hullegie, Darren Troeman, Denise van Hout, Daniël Prins, Rubana Kalyani, Mark Eickhoff, Kathryn Shoemaker, Tuba Vilken, Jelle Vlaeminck, Jasmine Coppens, Thomas van der Schalk, Basil Britto Xavier, Evelina Odisseeva, Rossitza Vatcheva, Michal Drab, Jaromir Vajter, Kadri Tamme, Muriel Fartoukh, Alain LePape, Mickael Landais, Gaetan Plantefève, Evelina Tacconelli, Achim Kaasch, Róbert Jurkinya, Iványi Zsolt, Miranda van Rijen, Olaf Cremer, Biljana Carevic, Jasna Jevdjić, Dolores Escudero, Miguel Sanchez Garcia, Cristina Prat-Aymerich, Borja Suberviola-Cañas, Angel Arenzana-Seisdedos, Hürrem Bodur, Cenk Kirakli, Ilkay Bozkurt, Sandra Long

**Affiliations:** 1 Center for Experimental and Molecular Medicine (CEMM), Amsterdam University Medical Centers, Location AMC, University of Amsterdam, Room G2-105, Meibergdreef 9, 1105 AZ Amsterdam, The Netherlands; 2 Julius Center for Health Sciences and Primary Care, University Medical Center Utrecht, Utrecht University, Utrecht, The Netherlands; 3 Laboratory of Medical Microbiology, Vaccine and Infectious Disease Institute, University of Antwerp, Antwerp, Belgium; 4 Department of Medical Microbiology, University Medical Center Utrecht, Utrecht University, Utrecht, The Netherlands

**Correction: Critical Care (2023) 27:269** 10.1186/s13054-023-04536-0

Following publication of the original article [1], the authors identified errors in the Additional file 1. A few of the supplementary figures were partially not visible.

The correct Additional file 1 file is included in this article.

Additional file 1 has been updated in this correction article and the original article [1] has been corrected.

### Supplementary Information

**Additional file 1**: STROBE statement; Supplementary methods: Definition of ICU-acquired pneumonia; Comorbidities; Causative pathogens; Sample collection; Assays; Statistical analysis; References; Supplementary tables: **Table S1.** Patient characteristics and clinical outcome of selected and not selected controls; **Table S2.** Missing clinical data; **Table S3.** Comorbidities; **Table S4.** Overview of linear mixed model estimates comparing cases with controls at baseline and onset of ICU-acquired pneumonia; **Table S5.** Patient characteristics and clinical outcome of cases in whom pneumonia occurred prior to day 7; **Table S6.** Patient characteristics of cases categorized into three groups according to the day of onset of ICU-acquired pneumonia; Supplementary figures: **Fig. S1.** Schematic representation of the clinical course of 277 cases during ICU-Stay; **Fig. S2.** Plasma protein biomarkers indicative of cytokine release and systemic inflammatory responses, and endothelial cell and procoagulant responses in Intensive Care Unit patients stratified according to the development of an ICU acquired Pneumonia or not at baseline and the time of the event. **Fig. S3.** Cytokine release and systemic inflammatory responses in ICU patients stratified according to the development of ICU-acquired pneumonia or not at baseline and event; **Fig. S4.** Endothelial cell and procoagulant responses in ICU patients stratified according to the development of ICU-acquired pneumonia or not at baseline and event; **Fig. S5.** Cytokine and systemic inflammatory responses in patients in whom pneumonia occurred prior to day 7; **Fig. S6.** Endothelial cell and procoagulant responses in Intensive Care Unit patients in whom pneumonia occurred prior to day 7; **Fig. S7.** Baseline cytokine release and systemic inflammatory responses in ICU patients who developed ICU acquired pneumonia; Fig. S8. Baseline endothelial cell and procoagulant responses in ICU patients who developed ICU-acquired pneumonia; ASPIRE-ICU Study Team.
