# Supplementary material for: Correction: Plasma protein biomarkers reflective of the host response in patients developing Intensive Care Unit-acquired pneumonia
Source: Crit Care. 2023 Nov 1;27:420. doi: 10.1186/s13054-023-04700-6 (PMC10621317; doi:10.1186/s13054-023-04700-6)
Supplement: Supplementary file 1 — Additional file 1: STROBE statement; Supplementary methods: Definition of ICU-acquired pneumonia; Comorbidities; Causative pathogens; Sample collection; Assays; Statistical analysis; References; Supplementary tables: Table S1. Patient characteristics and clinical outcome of selected and not selected controls; Table S2. Missing clinical data; Table S3. Comorbidities; Table S4. Overview of linear mixed model estimates comparing cases with controls at baseline and onset of ICU-acquired pneumonia; Table S5. Patient characteristics and clinical outcome of cases in whom pneumonia occurred prior to day 7; Table S6. Patient characteristics of cases categorized into three groups according to the day of onset of ICU-acquired pneumonia; Supplementary figures: Fig. S1. Schematic representation of the clinical course of 277 cases during ICU-Stay; Fig. S2. Plasma protein biomarkers indicative of cytokine release and systemic inflammatory responses, and endothelial cell and procoagulant responses in Intensive Care Unit patients stratified according to the development of an ICU acquired Pneumonia or not at baseline and the time of the event. Fig. S3. Cytokine release and systemic inflammatory responses in ICU patients stratified according to the development of ICU-acquired pneumonia or not at baseline and event; Fig. S4. Endothelial cell and procoagulant responses in ICU patients stratified according to the development of ICU-acquired pneumonia or not at baseline and event; Fig. S5. Cytokine and systemic inflammatory responses in patients in whom pneumonia occurred prior to day 7; Fig. S6. Endothelial cell and procoagulant responses in Intensive Care Unit patients in whom pneumonia occurred prior to day 7; Fig. S7. Baseline cytokine release and systemic inflammatory responses in ICU patients who developed ICU acquired pneumonia; Fig. S8. Baseline endothelial cell and procoagulant responses in ICU patients who developed ICU-acquired pneumonia; ASPIRE-ICU Study Team. [file 13054_2023_4700_MOESM1_ESM.pdf]

ADDITIONAL FILE 1

Plasma protein biomarkers reflective of the host response in patients developing Intensive Care Unit-acquired pneumonia

Tjitske S.R. van Engelen, Tom D.Y. Reijnders, Fleur P. Paling, Marc J.M. Bonten, Leen Timbermont, Surbhi Malhotra-Kumar, Jan A.J.W. Kluytmans, Hessel Peters-Sengers, Tom van der Poll; for ASPIRE-ICU Study Team

TABLE OF CONTENTS

STROBE STATEMENT 4
SUPPLEMENTARY METHODS 6
Definition of Intensive Care Unit-acquired pneumonia 6
Comorbidities 7
Causative pathogens 8
Sample collection 8
Assays 9
Statistical analysis 9
REFERENCES 11
SUPPLEMENTARY TABLES 12
Table S1. Patient characteristics and clinical outcome of selected and not selected controls 12
Table S2. Missing clinical data 13
Table S3. Comorbidities 14
Table S4. Overview of linear mixed model estimates comparing cases with controls at baseline and onset of Intensive Care Unit-acquired pneumonia 15
Table S5. Patient characteristics and clinical outcome of cases in whom pneumonia occurred prior to day 7 (i.e., in whom a baseline, event and post-pneumonia sample was obtained) 17
Table S6. Patient characteristics of cases categorized into three groups according to the day of onset of Intensive Care Unit-acquired pneumonia 18
SUPPLEMENTARY FIGURES 20
Fig. S1. Schematic representation of the clinical course of 277 cases during Intensive Care Unit-stay. 21
Fig. S2. Plasma protein biomarkers indicative of cytokine release and systemic inflammatory responses, and endothelial cell and procoagulant responses in Intensive Care Unit (ICU) patients stratified according to the development of an ICU-acquired pneumonia (case) or not (control) at baseline and the time of the event 22
Fig. S3. Cytokine release and systemic inflammatory responses in Intensive Care Unit (ICU) patients stratified according to the development of ICU-acquired pneumonia (cases, red) or not (controls, blue) at baseline and event. 23
Fig. S4. Endothelial cell and procoagulant responses in Intensive Care Unit (ICU) patients stratified according to the development of ICU-acquired pneumonia (cases, red) or not (controls, blue) at baseline and event. 24
Fig. S5. Cytokine and systemic inflammatory responses in patients in whom pneumonia occurred prior to day 7 (i.e., in whom a baseline, event and post-pneumonia sample was obtained) 25

Fig. S6. Endothelial cell and procoagulant responses in Intensive Care Unit (ICU) patients in whom pneumonia occurred prior to day 7 (i.e., in whom a baseline, event and post-pneumonia sample was obtained) 26

Fig. S7. Baseline cytokine release and systemic inflammatory responses in Intensive Care Unit (ICU) patients who developed ICU-acquired pneumonia. 27

Fig. S8. Baseline endothelial cell and procoagulant responses in Intensive Care Unit (ICU) patients who developed ICU-acquired pneumonia. 28

ASPIRE-ICU STUDY TEAM 29

STROBE STATEMENT

	Item	Recommendation	Page
**Title and abstract**	1	(*a*) Indicate the study's design with a commonly used term in the title or the abstract	1
		(*b*) Provide in the abstract an informative and balanced summary of what was done and what was found	1
**Introduction**			
Background/rationale	2	Explain the scientific background and rationale for the investigation being reported	3
Objectives	3	State specific objectives, including any prespecified hypotheses	3
**Methods**			
Study design	4	Present key elements of study design early in the paper	4
Setting	5	Describe the setting, locations, and relevant dates, including periods of recruitment, exposure, follow-up, and data collection	4
Participants	6	(*a*) Give the eligibility criteria, and the sources and methods of selection of participants. Describe methods of follow-up	4
		(*b*) For matched studies, give matching criteria and number of exposed and unexposed	NA
Variables	7	Clearly define all outcomes, exposures, predictors, potential confounders, and effect modifiers. Give diagnostic criteria, if applicable	4-5, Additional File 1: 7-9
Data sources/ measurement	8	For each variable of interest, give sources of data and details of methods of assessment (measurement). Describe comparability of assessment methods if there is more than one group	5-6, Additional File 1: 9-11
Bias	9	Describe any efforts to address potential sources of bias	5
Study size	10	Explain how the study size was arrived at	4
Quantitative variables	11	Explain how quantitative variables were handled in the analyses. If applicable, describe which groupings were chosen and why	4-5, Additional File 1: 10-11
Statistical methods	12	(*a*) Describe all statistical methods, including those used to control for confounding	5, Additional File 1: 10-11
		(*b*) Describe any methods used to examine subgroups and interactions	5, Additional File 1: 10-11
		(*c*) Explain how missing data were addressed	5, Additional File 1: 10, 14
		(*d*) If applicable, explain how loss to follow-up was addressed	NA
		(*e*) Describe any sensitivity analyses	NA
**Results**			
Participants	13	(a) Report numbers of individuals at each stage of study—eg numbers potentially eligible, examined for eligibility, confirmed eligible, included in the study, completing follow-up, and analyzed	5-6, Fig. 1
		(b) Give reasons for non-participation at each stage	6, Fig. 1
		(c) Consider use of a flow diagram	Fig.1
Descriptive data	14*	(a) Give characteristics of study participants (eg demographic, clinical, social) and information on exposures and potential confounders	5-6, Additional File 1: 13, 15, 18-19
		(b) Indicate number of participants with missing data for each variable of interest	5-6, Additional File 1: 14

		(c) Summarise follow-up time (eg, average and total amount)	5-6, Additional File 1: 22
Outcome data	15	Report numbers of outcome events or summary measures over time	6-9
Main results	16	(*a*) Give unadjusted estimates and, if applicable, confounder-adjusted estimates and their precision (eg, 95% confidence interval). Make clear which confounders were adjusted for and why they were included	6-9, Additional File 1: 16-17
		(*b*) Report category boundaries when continuous variables were categorized	6-9
		(*c*) If relevant, consider translating estimates of relative risk into absolute risk for a meaningful time period	NA
Other analyses	17	Report other analyses done—eg analyses of subgroups and interactions, and sensitivity analyses	8-9, Additional File 1: 16-19, 26-29
**Discussion**			
Key results	18	Summarise key results with reference to study objectives	9
Limitations	19	Discuss limitations of the study, taking into account sources of potential bias or imprecision. Discuss both direction and magnitude of any potential bias	11
Interpretation	20	Give a cautious overall interpretation of results considering objectives, limitations, multiplicity of analyses, results from similar studies, and other relevant evidence	9-11
Generalisability	21	Discuss the generalisability (external validity) of the study results	11
**Other information**			
Funding	22	Give the source of funding and the role of the funders for the present study and, if applicable, for the original study on which the present article is based	15

SUPPLEMENTARY METHODS

Definition of Intensive Care Unit-acquired pneumonia

ICU pneumonia in mechanically ventilated patients

Patient should demonstrate the following new onset of symptoms/signs deemed not due to any overt non-infectious causes.

a. Radiographic criteria:

New or worsening infiltrate consistent with pneumonia on chest X-ray or CT-thorax obtained within 24 hours of the event (diagnosed by a qualified radiologist).

AND

b. Clinical criteria:

At least **2** of the following minor or **1** major respiratory sign or symptom of new onset:

Minor criteria:

- Systemic signs of infection (one or more of the following): Abnormal temperature (oral or tympanic temperature > 38°C or a core temperature ≥ 38.3°C or hypothermia, defined as a core body temperature of < 35°C), and/or abnormal WBC (WBC count > 10,000 cells/mm^3, WBC count < 4500 cells/mm^3, or > 15% band neutrophils)
- Production of purulent endotracheal secretions
- Auscultatory findings consistent with pneumonia/pulmonary consolidation (e.g. rales, rhonchi, bronchial breath sounds, dullness to percussion)

Major criteria: Acute changes made in the ventilatory support system to enhance oxygenation, as determined by:

- PaO_2/FiO_2 ratio < 240 mmHg, or
- A decrease in PaO_2/FiO_2 by ≥ 50 mmHg

ICU pneumonia in not mechanically ventilated patients

Patient should demonstrate the following new onset of symptoms/signs deemed not due to any overt non-infectious causes.

a. Radiographic criteria:

New or worsening infiltrate consistent with pneumonia on chest X-ray or CT-thorax obtained within 24 hours of the event (diagnosed by qualified radiologist)

AND

b. Clinical criteria:

At least **2** of the following minor or **1** major respiratory signs or symptoms:

Minor criteria:

- Systemic signs of infection: Abnormal temperature (oral or tympanic temperature > 38°C or a core temperature ≥ 38.3°C or hypothermia, defined as a core body temperature of < 35°C), and/or abnormal WBC (WBC count > 10,000 cells/mm^3, WBC count < 4500 cells/mm^3, or > 15% band neutrophils)
- A new onset of cough (or worsening of cough)
- Production of purulent sputum

- Physical examination findings consistent with pneumonia/pulmonary consolidation such as auscultatory findings (e.g. rales, rhonchi, bronchial breath sounds), dullness to percussion, or pleuritic chest pain
- Dyspnea, tachypnea (respiratory rate > 30 breaths/minute), or hypoxemia defined as:
  - O_2 saturation < 90% or PaO_2 < 60 mmHg on room air if lower than baseline, or
  - A need to initiate or increase sustained (≥ 3 hours) supplemental oxygen to maintain pre-event baseline O_2 saturations

Major criteria:

A need to initiate non-invasive mechanical ventilation or re-initiate invasive mechanical ventilation because of respiratory failure or worsening of respiratory status

Comorbidities

Study definitions for the comorbidities at ICU admission	
Myocardial infarction	History of medically documented myocardial infarction, not ECG changes only.
Congestive heart failure	Symptomatic congestive heart failure (exertional or paroxysmal nocturnal dyspnea) with response to specific treatment (digitalis, diuretics, or afterload reducing agents).
Peripheral vascular disease	Intermittent claudication, peripheral arterial bypass for insufficiency, gangrene, acute arterial insufficiency, untreated aneurysm (≥ 6 cm)
Cerebrovascular accident	History of transient ischaemic attack, or cerebrovascular accident with no or minor sequelae.
Dementia	Chronic cognitive deficit prior to this hospital admission.
Chronic pulmonary disease	Moderate: dyspnoeic with slight activity, with or without treatment, and dyspnoeic with moderate activity despite treatment. Severe: dyspnoeic at rest, despite treatment, requires constant oxygen; CO2 retention and a baseline PO2 below 50 torr.
Connective tissue disease	Diagnosis of either systemic lupus erythematosus, polymyositis, mixed connective tissue disease, polymyalgia rheumatica, moderate to severe rheumatoid arthritis.
Peptic ulcer disease	Patients who required prior treatment for peptic ulcer disease, including bleeding from ulcers.
Renal disease	Moderate: serum creatinine > 3 mg/dL (or >265μmol/L). Severe: on dialysis, had a transplant, and with uremia.
Mild liver disease	Cirrhosis without portal hypertension or chronic hepatitis.
Moderate to severe liver disease	Moderate: cirrhosis with portal hypertension, but without history of variceal bleeding. Severe: cirrhosis, portal hypertension, and a history of variceal bleeding.
Diabetes without end-organ damage	Mild: diabetes treated with insulin or oral hypoglycemics (not with diet alone). Moderate: includes if a subject had previous hospitalizations for ketoacidosis, hyperosmolar coma, or/and those with juvenile onset or brittle diabetics.
Diabetes with end-organ damage	Diabetes with either retinopathy, neuropathy, nephropathy.
Hemiplegia	Hemiplegia or paraplegia, as a result of either a cerebrovascular accident or other conditions.
Leukemia	Diagnosis or prior diagnosis of either acute myeloid leukemia, chronic myeloid leukemia, acute lymphocytic

	leukemia, chronic lymphocytic leukemia, and polycythemia vera.
Lymphoma	Diagnosis or prior diagnosis of either Hodgkin's disease, lymphosarcoma, Waldenstrom macroglobulinemia, myeloma, and other lymphomas.
Solid tumor without metastasis	Solid tumors without documented metastases, but initially treated in the last 5 years.
Solid tumor with metastasis	Solid tumor with metastases.
Acquired Immune Deficiency Syndrome (AIDS)	Definite or probable AIDS (i.e., AIDS related complex). AIDS is defined as a HIV+ status with a CD4 cell count <200 cells/microLiter or the presence of any AIDS-defining condition regardless of the CD4 cell count. AIDS-defining conditions are opportunistic illnesses that occur more frequently or more severely because of immunosuppression. These include mainly opportunistic infections, but also certain malignancies as well as conditions without clear alternative etiology thought to be related to uncontrolled HIV infection itself, such as wasting or encephalopathy.
Immunosuppression	The subject has received therapy that suppresses resistance to infection e.g. immunosuppressants, chemotherapy, radiation, long term or recent high dose steroids*, or has a disease that is sufficiently advanced to suppress.<br>*The following is considered high dose of corticosteroids: If a subject uses one or more of the following medications daily for≥14 days intravenous or oral (so don't include 'every other day' or topical use): prednisone ≥ 20mg (Lodotra, Di-Adreson-F), methylprednisolone≥16mg (Depo-Medrol/Solu-Medrol), cortisone≥100mg, hydrocortisone≥80mg (Solu-Cortef), dexamethasone≥3,2mg (Oradexon), betamethasone≥3,2mg (Celestone), triamcinolone(acetamide) ≥16mg (Kenacort-A).

Adapted from [1].

Causative pathogens

Causative pathogens were determined post hoc based on isolation of a respiratory pathogen from any lower respiratory tract specimen (including both clinical and study surveillance cultures) or blood culture in the 3 days before and after the day of pneumonia diagnosis [2, 3].

Sample collection

Ethylenediaminetetraacetic acid (EDTA) anticoagulated blood samples were centrifuged at the local site at 1500 g for 15 minutes (preferably at 4°C), after which plasma was transferred into plasma storage tubes and stored at -70°C as soon as possible and within 3 hours after sample collection at the ICU. Samples were regularly transferred from the local sites to the central laboratory at the University of Antwerp, Belgium, and further transferred in four batches to the Amsterdam UMC biobank, the Netherlands, all on dry ice. Normal reference values were obtained

in plasma collected from 19 age and sex matched subjects after having provided written informed consent (part of the ELDER-BIOME study, clinicaltrials.gov identifier NCT02928367).

Assays

We measured 19 host response biomarkers on a BioPlex-machine (Bio-Plex 200, Bio-Rad, Hercules, CA, USA) using a custom-made Luminex multiplex assay (R&D Systems Inc, Minneapolis, MN, USA). All samples were diluted 1:2 in assay buffer and all biomarkers were in one assay. We categorized these biomarkers into four pathophysiological domains: interleukin (IL)-6, IL-8, IL-10, and IL-1 receptor antagonist (IL-1RA)(reflecting cytokine release); matrix metalloproteinase (MMP)-8, soluble triggering receptor expressed on myeloid cells (sTREM)-1, soluble cluster of differentiation (sCD)163, soluble receptor for advanced glycation endproducts (sRAGE), tenascin-C and procalcitonin (reflecting systemic inflammation); sE-selectin, soluble vascular cell adhesion protein (sVCAM)-1, fractalkine, syndecan-1, soluble thrombomodulin, angiopoietin-1, and angiopoietin-2 (reflecting endothelial activation and function); soluble tissue factor and D-dimer (reflecting coagulation activation). Samples were randomly distributed over assay plates stratified per subgroup of interest (i.e. case baseline, case event, case day 7, control baseline, control day 7, and healthy subjects), such that the proportion of each subgroup on each plate was the same, and the central tendency and spread of each biomarker on each plate was expected to be the same. We removed individual analytes if fewer than 25 beads were measured for that analyte in that sample.

Batch effects (non-biological and non-random variation in biomarker levels) between plates were corrected using multiple bridging samples present on each plate in duplicate. We fitted linear regression models per analyte to obtain scaling factors for each plate relative to the first plate of the measurement day. Biomarker measurements below the limit of quantification were imputed as half the lower limit of quantification. This was necessary for 418/1796 [23.3%] of IL-10 measurements, but only 9/32328 (0.03%) of all other biomarker measurements. We accepted extrapolated biomarker values up to ten times the upper limit of quantification. Measurements higher than ten times the upper limit of quantification (in 115/34124 [0.34%] total data points) were set to the upper limit of quantification.

Statistical analysis

Statistical analysis was performed in the R statistical framework (Version 4.2.0, R Core Team 2020. R: A language and environment for statistical computing. R Foundation for Statistical Computing, Vienna, Austria); the package NLME (Linear and Nonlinear Mixed Effects Models) was used for linear mixed model analyses, and the packages ggeffects with ggplot2 for visual purposes. After $\log_{10}$ transformation of biomarker values, regression coefficients with 95% confidence intervals were backtransformed. Mixed model analyses were used to compare host response differences between cases and controls at baseline, host response biomarkers at the time of ICU-acquired pneumonia diagnosis relative to controls, and host response trajectories, i.e. the change in biomarkers over time from prior to ICU-acquired pneumonia to the day of ICU-acquired pneumonia. The compound symmetry structure was chosen as variance-covariance matrix which formally outperformed the goodness of fit indices (i.e. -2*log

likelihood ratio test and subtracting Akaike's information criterion) of other common chosen correlation structures (i.e. autoregressive process of order 1, and unstructured correlation matrix).

For 16 patients in the case group in whom the sample drawn on day 7 after inclusion was taken on the same day as pneumonia was diagnosed, both samples were treated as event sample in the mixed model analyses. In cases in whom the day 7 sample was taken after the event (i.e., in these patients ICU-acquired pneumonia was diagnosed prior to day 7; eTable 5) biomarker trajectories across three time points were included in the mixed model without controls. Differences between time points were analyzed by contrast dummy coding of the time variable and by changing the reference category.

In another analysis only including cases, linear regression was used with log-transformed biomarkers at baseline as outcome and time to ICU-acquired pneumonia in days as predictor. Time cut-offs to onset of ICU-acquired pneumonia (0-5 days, 6-9 days, >10 days) were chosen to maintain a balanced number of patients in each group (eTable 6). Nonlinearity was assessed by using restricted cubic splines with 3 predefined internal knots at day 3, 6, and 10. Expanding the number of knots did not improve the model fit of Akaike's information criterion.

All results are presented as counts (percentages) for categorical variables, median and interquartile ranges (IQR, 25th and 75th percentiles) for nonparametric quantitative variables, and mean ± standard deviation of the mean (SD) for parametric quantitative variables. Histograms, density plots and Q-Q plots were examined to assess data distribution. Continuous nonparametric data were analyzed using a Wilcoxon signed rank sum- or Kruskal-Wallis test; categorical data were analyzed using a Fisher exact test; continuous parametric data were analyzed using a Student t test. Group differences of the biomarker results were corrected for multiple testing using the Benjamini-Hochberg method. Moderation effects were not corrected for multiple testing. *P* values below 0.05 were considered statistically significant.

REFERENCES

1. Charlson ME, Pompei P, Ales KL, MacKenzie CR. A new method of classifying prognostic comorbidity in longitudinal studies: development and validation. *J Chronic Dis* 1987;40:373–383. https://doi.org/10.1016/0021-9681(87)90171-8

2. Paling FP, Troeman DPR, Wolkewitz M, Kalyani R, Prins DR, Weber S, et al. Rationale and design of ASPIRE-ICU: a prospective cohort study on the incidence and predictors of Staphylococcus aureus and Pseudomonas aeruginosa pneumonia in the ICU. *BMC Infect Dis* 2017;17:643. https://doi.org/10.1186/s12879-017-2739-4

3. Paling FP, Hazard D, Bonten MJM, Goossens H, Jafri HS, Malhotra-Kumar S, et al. Association of Staphylococcus aureus Colonization and Pneumonia in the Intensive Care Unit. *JAMA Netw open* 2020;3:e2012741. https://doi.org/10.1001/jamanetworkopen.2020.12741

SUPPLEMENTARY TABLES

Table S1. Patient characteristics and clinical outcome of selected and not selected controls

	**Selected controls** (*n* = 632)	**Not selected controls** (*n* = 1049)	***P* value**
Baseline data			
Age, yr, mean (SD)	63 (16.0)	62 (16.0)	0.48
Female sex, n (%)	227 (35.9)	379 (36.1)	0.97
Body Mass Index, mean (SD)	27.2 (6.0)	27.3 (6.3)	0.73
Charlson Comorbidity Index, median (IQR)	3 (2–5)	3 (1–5)	0.31
Origin prior to ICU admission, n (%)			0.17
Home	326 (51.6)	569 (54.2)	
General ward	178 (28.2)	305 (29.1)	
Long term facility	14 (2.2)	10 (1.0)	
Other ICU	97 (15.3)	139 (13.3)	
Primary reason for ICU admission, n (%)			0.19
Medical	292 (46.2)	542 (51.7)	
Surgical, cardiothoracic	44 (7.0)	69 (6.6)	
Surgical, other	171 (27.1)	250 (23.8)	
Trauma	125 (19.8)	188 (17.9)	
Colonized with *Staphylococcus aureus* on admission, n (%)	316 (50.0)	519 (49.5)	0.88
Pneumonia on ICU admission, n (%)	124 (19.6)	191 (18.3)	0.53
APACHE IV score, mean (SD)	70 (38.0)	73 (37.9)	0.17
Laboratory values at ICU admission			
White blood cells, 10^9/L, median (IQR)	13.0 (9.2–17.5)	13.2 (9.0–18.4)	0.83
Neutrophils, 10^9/L, median (IQR)	11.0 (7.2–15.2)	10.9 (6.7–15.7)	0.94
Monocytes, 10^9/L, median (IQR)	0.7 (0.4–1.1)	0.7 (0.4–1.0)	0.33
Lymphocytes, 10^9/L, median (IQR)	0.8 (0.5–1.3)	0.9 (0.5–1.5)	0.39
Platelets, 10^9/L, median (IQR)	205 (148–271)	201 (146–271)	0.75
Outcome data			
Length of ICU stay, days, median (IQR)	8 (5–14)	7 (5–14)	0.57
Readmission <30 days of ICU discharge, n (%)	21 (3.3)	57 (5.4)	0.11
Status at Day 90 after ICU admission, n (%)			0.85
Alive	368 (58.2)	603 (57.5)	
Dead	208 (32.9)	354 (33.7)	
Unknown or missing	56 (8.9)	92 (8.8)	

APACHE IV Acute Physiology and Chronic Health Evaluation IV; *ICU* intensive care unit; *IQR* interquartile range. A control was defined as a subject who did not develop a protocol defined ICU-acquired pneumonia. From all controls (n=1681) we randomly selected a subset in a 2:1 ratio to the cases using the sample function without replacement of the R statistical framework. Continuous nonparametric data were analyzed using a Wilcoxon signed rank sum- or Kruskal-Wallis test; categorical data were analyzed using a Fisher exact test; continuous parametric data were analyzed using a Student t test; a *P* value < 0.05 was considered statistically significant.

Table S2. Missing clinical data

	**Cases** (*n* = 277)	**Controls** (*n* = 632)
Baseline data		
Age	0	0
Female sex	0	0
Body Mass Index	4 (1.4)	7 (1.1)
Charlson Comorbidity Index	0	1 (0.2)
Origin prior to ICU admission	11 (4.0)	17 (2.7)
Primary reason for ICU admission	0	0
Colonized with *Staphylococcus aureus* on admission	0	0
Pneumonia on ICU admission	0	0
APACHE IV score	0	0
Laboratory values at ICU admission		
White blood cells	1 (0.4)	6 (0.9)
Neutrophils	118 (42.6)	220 (34.8)
Monocytes	129 (46.6)	232 (36.7)
Lymphocytes	114 (41.2)	217 (34.3)
Platelets	2 (0.7)	6 (0.9)
Outcome data		
Length of ICU stay	0	0
Readmission <30 days of ICU discharge	0	0
Status at Day 90 after ICU admission	21 (7.6)	56 (8.9)

APACHE IV Acute Physiology and Chronic Health Evaluation IV; *ICU* intensive care unit; *IQR* interquartile range. Variables are displayed as count (percentage).

Table S3. Comorbidities

	**Cases** (*n* = 316)	**Controls** (*n* = 632)	***P* value**
Myocardial infarction	33 (10.4)	67 (10.6)	>0.99
Congestive heart failure	38 (12.0)	83 (13.2)	0.70
Peripheral vascular disease	19 (6.0)	43 (6.8)	0.74
Cerebrovascular accident	26 (8.2)	66 (10.5)	0.33
Dementia	5 (1.6)	19 (3.0)	0.27
Chronic pulmonary disease	64 (20.3)	112 (17.7)	0.40
Connective tissue disease	7 (2.2)	11 (1.7)	0.80
Peptic ulcer disease	9 (2.8)	28 (4.4)	0.31
Renal disease	33 (10.4)	55 (8.7)	0.46
Mild liver disease	1 (0.3)	22 (3.5)	0.01
Moderate to severe liver disease	7 (2.2)	17 (2.7)	0.82
Diabetes without end-organ damage	47 (14.9)	88 (13.9)	0.78
Diabetes with end-organ damage	15 (4.7)	50 (7.9)	0.09
Hemiplegia	8 (2.5)	21 (3.3)	0.64
Leukemia	4 (1.3)	2 (0.3)	0.19
Lymphoma	4 (1.3)	7 (1.1)	>0.99
Solid tumor without metastasis	33 (10.4)	62 (9.8)	0.85
Solid tumor with metastasis	13 (4.1)	24 (3.8)	0.96
Acquired Immune Deficiency Syndrome	2 (0.6)	2 (0.3)	0.86
Immunosuppression	25 (7.9)	28 (4.4)	0.04

Data are n (%). Continuous nonparametric data were analyzed using a Wilcoxon signed rank sum- or Kruskal-Wallis test; categorical data were analyzed using a Fisher exact test; continuous parametric data were analyzed using a Student t test; a *P* value < 0.05 was considered statistically significant.

Table S4. Overview of linear mixed model estimates comparing cases with controls at baseline and onset of Intensive Care Unit-acquired pneumonia

		**Baseline Group difference**		**Event Group difference**		**Interaction**
		coefficient (95%CI)	*P* value (BH)	coefficient (95%CI)	*P* value (BH)	*P* value
Cytokine release and systemic inflammation						
IL-6	unadjusted	48.0% (22.6—78.6)	<0.0001	49.7% (59.4—37.7)	<0.0001	0.0109
	adjusted	46.3% (21.3—76.5)	0.0014	49.1% (58.9—37)	<0.0001	0.0110
IL-8	unadjusted	12.5% (-1.6—28.5)	0.1229	19.4% (30.7—6.2)	0.0090	0.2169
	adjusted	10.6% (-3.1—26.2)	0.2024	17.6% (29.1—4.3)	0.0183	0.2364
IL10	unadjusted	10.3% (-1.5—23.6)	0.1229	9.0% (20.2—3.7)	0.1842	0.9615
	adjusted	9.0% (-2.8—22.2)	0.2024	8.0% (19.3—4.8)	0.2472	0.9739
IL-1RA	unadjusted	26.6% (7.1—49.7)	0.0220	26.5% (39.3—11)	0.0033	0.4960
	adjusted	24.9% (6.0—47.3)	0.0306	25.7% (38.4—10.4)	0.0047	0.4803
Procalcitonin	unadjusted	50.2% (18—91.3)	0.0062	53.1% (64.2—38.5)	<0.0001	0.0099
	adjusted	46.3% (15.9—84.6)	0.0087	51.4% (62.6—36.9)	<0.0001	0.0112
MMP-8	unadjusted	48.1% (20.7—81.8)	0.0017	43.2% (55.2—28.1)	<0.0001	0.1596
	adjusted	45.2% (18.4—78.2)	0.0034	41.6% (53.9—26.1)	<0.0001	0.1806
sTREM-1	unadjusted	12.5% (2.2—23.8)	0.0351	15.9% (24.3—6.7)	0.0027	0.1827
	adjusted	9.7% (0.5—19.7)	0.0935	13.5% (21.4—4.8)	0.0066	0.2050
sRAGE	unadjusted	17.1% (6.3—29)	0.0069	25.5% (33.1—17)	<0.0001	0.0059
	adjusted	15% (4.8—26.3)	0.0158	24.2% (31.6—15.9)	<0.0001	0.0057
Tenascin-C	unadjusted	3.8% (-3—11.1)	0.2954	11.4% (17.8—4.4)	0.0033	0.0177
	adjusted	2.4% (-4.2—9.5)	0.5043	10.2% (16.6—3.3)	0.0087	0.0174
sCD163	unadjusted	8.3% (-6.1—25)	0.2954	8.4% (21.6—7.0)	0.2671	0.9030
	adjusted	5.4% (-8.2—21.1)	0.5043	5.4% (18.7—10.0)	0.4695	0.9597

		Baseline Group difference		Event Group difference		Interaction
		coefficient (95%CI)	*P* value (BH)	coefficient (95%CI)	*P* value (BH)	*P* value
Endothelial cell and procoagulant response						
sE-selectin	unadjusted	6.8% (-3.4—18)	0.2383	13.5% (22.5—3.4)	0.0148	0.0992
	adjusted	6.1% (-3.9—17.1)	0.2846	13.1% (22—3.1)	0.0183	0.0925
sVCAM-1	unadjusted	15.7% (-0.5—34.4)	0.0997	26.1% (37.5—12.7)	0.0012	0.0456
	adjusted	11.8% (-3.5—29.4)	0.2024	23% (34.6—9.4)	0.0046	0.0544
Fractalkine	unadjusted	6.5% (1.3—12)	0.0334	9.6% (14.6—4.3)	0.0014	0.1874
	adjusted	5.8% (0.6—11.2)	0.0750	9% (14—3.7)	0.0036	0.1886
s-Thrombomodulin	unadjusted	8.0% (-0.1—16.8)	0.0996	9.8% (17—1.9)	0.0221	0.4162
	adjusted	5.3% (-2.1—13.2)	0.2265	7.4% (14.4—0.2)	0.0757	0.4163
Syndecan-1	unadjusted	8.3% (-2.7—20.6)	0.1830	6.7% (17.1—5.1)	0.2671	0.8356
	adjusted	6.7% (-3.6—18.2)	0.2681	4.8% (15—6.6)	0.4348	0.7617
Angiopoietin-1	unadjusted	-3.7% (-18.3—13.5)	0.6517	-11.6% (-34.2—7.2)	0.2671	0.4243
	adjusted	0.1% (-14.8—17.6)	0.9895	-7.9% (-29.2—10.0)	0.4348	0.3954
Angiopoietin-2	unadjusted	17.7% (4—33.1)	0.0308	33.8% (42.3—24.1)	<0.0001	<0.0001
	adjusted	16.4% (3.1—31.3)	0.0444	32.8% (41.3—23.1)	<0.0001	<0.0001
s-Tissue factor	unadjusted	10.1% (2—18.8)	0.0334	10.8% (17.9—3.1)	0.0108	0.5754
	adjusted	6.5% (-0.8—14.3)	0.1534	8% (14.8—0.6)	0.0506	0.5409
D-dimer	unadjusted	8.6% (-0.7—18.8)	0.1116	7.4% (16.3—2.5)	0.1773	0.9023
	adjusted	8.4% (-0.8—18.3)	0.1534	7.3% (16.1—2.4)	0.1702	0.9348

BH Benjamini Hochberg adjustment for multiple testing. The coefficient represents a relative change of the plasma biomarker concentration of cases compared to controls. All biomarkers were log-transformed, and backtransformed for interpretability to obtain differences between cases and controls in %. Interaction indicates if there is a different biomarker trajectory from baseline to onset of ICU-acquired pneumonia for cases as compared to controls (not corrected for multiple testing). Models were adjusted for following potential confounders: site of enrollment, age, sex, body mass index, primary reason for ICU admission, Charlson Comorbidity Index, immunodeficient as chronic comorbidity, Acute Physiology and Chronic Health Evaluation IV score.

Table S5. Patient characteristics and clinical outcome of cases in whom pneumonia occurred prior to day 7 (i.e., in whom a baseline, event and post-pneumonia sample was obtained)

	**Overall** (*n* = 122)
Baseline data	
Age, yr, mean (SD)	62.3 (16.1)
Female sex, n (%)	90 (73.8)
Body Mass Index, mean (SD)	26.7 (5.1)
Charlson Comorbidity Index, median (IQR)	3.0 [2.0, 5.0]
Origin prior to ICU admission, n (%)	
Home	33 (27.0)
General ward	66 (54.1)
Long term facility	19 (15.6)
Other ICU	4 (3.3)
Primary reason for ICU admission, n (%)	
Medical	51 (41.8)
Surgical, cardiothoracic	5 (4.1)
Surgical, other	38 (31.1)
Trauma	28 (23.0)
Colonized with Staphylococcus aureus on admission, n (%)	63 (51.6)
Pneumonia on ICU admission, n (%)	17 (13.9)
APACHE IV, mean (SD)	70.7 (38.1)
Laboratory values at ICU admission	
White blood cells, 10^9/L, median (IQR)	13.3 [9.4, 19.5]
Neutrophils, 10^9/L, median (IQR)	10.7 [7.1, 14.8]
Monocytes, 10^9/L, median (IQR)	0.7 [0.5, 1.0]
Lymphocytes, 10^9/L, median (IQR)	0.9 [0.5, 1.2]
Platelets, 10^9/L, median (IQR)	201.5 [151.5, 257.2]
Outcome data	
Length of ICU stay (median [IQR])	14.5 [11.0, 23.8]
Readmission <30 days of ICU discharge, n (%)	8 (6.6)
90-day mortality after ICU admission, n (%)	45 (39.8)

APACHE IV Acute Physiology and Chronic Health Evaluation IV; *ICU* intensive care unit; *IQR* interquartile range. Continuous nonparametric data were analyzed using a Wilcoxon signed rank sum- or Kruskal-Wallis test; categorical data were analyzed using a Fisher exact test; continuous parametric data were analyzed using a Student t test.

Table S6. Patient characteristics of cases categorized into three groups according to the day of onset of Intensive Care Unit-acquired pneumonia

	**ICU-A pneumonia 0-5 days** (*n* = 105)	**ICU-A pneumonia 6-9 days** (*n* = 69)	**ICU-A pneumonia >10 days** (*n* = 68)	***P* value**
Baseline data				
Age, yr, mean (SD)	63.7 (16.6)	62.0 (14.1)	64.7 (15.4)	0.585
Female sex, n (%)	67 (63.8)	56 (81.2)	44 (64.7)	0.035
Body Mass Index, mean (SD)	26.8 (5.9)	27.0 (4.7)	28.3 (7.9)	0.282
Charlson Comorbidity Index, median (IQR)	3.0 [2.0, 5.0]	3.0 [1.0, 4.0]	4.0 [2.0, 5.0]	0.285
Origin prior to ICU admission, n (%)				0.352
Home	63 (60.0)	33 (47.8)	32 (47.1)	
General ward	27 (25.7)	19 (27.5)	22 (32.4)	
Long term facility	0 (0.0)	0 (0.0)	1 (1.5)	
Other ICU	12 (11.4)	13 (18.8)	12 (17.6)	
Other	3 (2.9)	4 (5.8)	1 (1.5)	
Primary reason for ICU admission, n (%)				0.928
Medical	50 (47.6)	33 (47.8)	33 (48.5)	
Surgical, cardiothoracic	3 (2.9)	2 (2.9)	1 (1.5)	
Surgical, other	30 (28.6)	17 (24.6)	22 (32.4)	
Trauma	22 (21.0)	17 (24.6)	12 (17.6)	
Colonized with *Staphylococcus aureus* on admission, n (%)	60 (57.1)	34 (49.3)	35 (51.5)	0.559
Pneumonia on ICU admission, n (%)	17 (16.2)	11 (15.9)	18 (26.5)	0.181
APACHE IV, mean (SD)	76.0 (37.2)	65.2 (37.0)	76.1 (34.7)	0.116
Laboratory values at ICU admission				
White blood cells, 10^9/L, median (IQR)	13.2 [9.0, 19.5]	13.3 [9.8, 18.0]	11.4 [7.8, 15.8]	0.097
Neutrophils, 10^9/L, median (IQR)	11.3 [7.4, 14.9]	12.1 [8.2, 14.3]	10.2 [6.9, 13.6]	0.184
Monocytes, 10^9/L, median (IQR)	0.7 [0.5, 1.0]	0.7 [0.6, 1.1]	0.5 [0.3, 0.9]	0.031
Lymphocytes, 10^9/L, median (IQR)	1.0 [0.6, 1.3]	0.9 [0.5, 1.2]	0.7 [0.5, 0.9]	0.040
Platelets, 10^9/L, median (IQR)	208.0 [157.0, 258.0]	202.0 [151.0, 268.0]	173.0 [118.0, 246.0]	0.041
Outcome data				
Length of ICU stay (median [IQR])	13.0 [9.0, 20.0]	14.0 [10.0, 23.0]	32.0 [23.8, 40.0]	<0.001
Readmission <30 days of ICU discharge, n (%)	6 (5.7)	2 (2.9)	3 (4.4)	0.495
90-day mortality after ICU admission, n (%)	43 (43.9)	25 (39.7)	35 (55.6)	0.173

APACHE IV Acute Physiology and Chronic Health Evaluation IV; *ICU* intensive care unit; *IQR* interquartile range. A case was defined as a subject who developed a (by protocol defined) ICU-acquired pneumonia. Continuous nonparametric data were analyzed using a Wilcoxon signed rank sum- or Kruskal-Wallis test; categorical data were analyzed using a Fisher exact test; continuous parametric data were analyzed using a Student t test; a *P* value < 0.05 was considered statistically significant.

SUPPLEMENTARY FIGURES

Fig. S1. Schematic representation of the clinical course of 277 cases during Intensive Care Unit-stay.

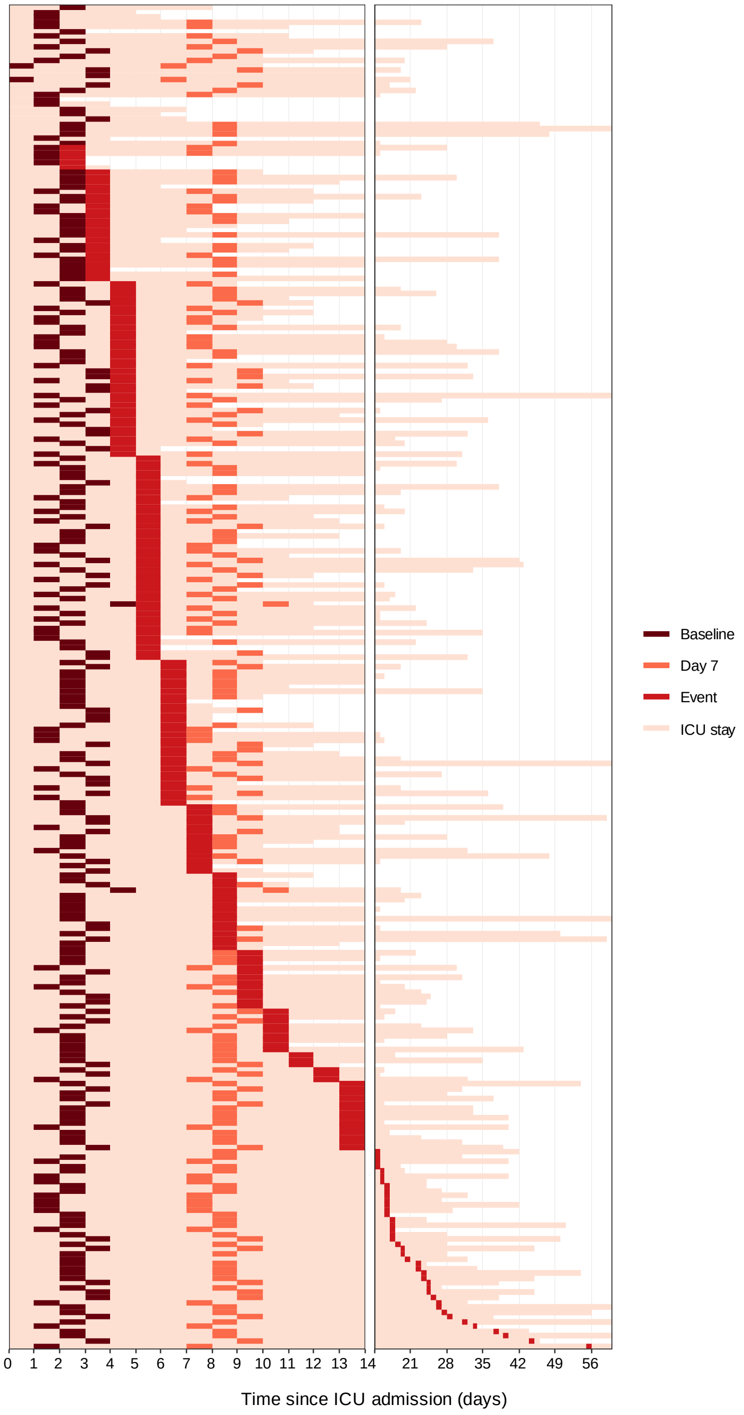

Each bar represents a case with its individual length of Intensive Care Unit (ICU)-stay and colours indicate sampling timepoints. Blood was obtained upon enrolment into ASPIRE-ICU (baseline), on the day the pneumonia was diagnosed (event), and on day 7 after study inclusion. There was no event-sample available for analysis in 29 cases. In 16 cases the sample drawn on day 7 after inclusion was taken on the same day as pneumonia was diagnosed. In 135 cases (54.9%) the event was prior to the standard follow-up sample taken 7 days after enrolment into the study; of these, from 122 were 3 sequential samples (baseline, event, post-event) available for biomarker analyses. *Definition of abbreviations:* ICU = intensive care unit.

Fig. S2. Plasma protein biomarkers indicative of cytokine release and systemic inflammatory responses, and endothelial cell and procoagulant responses in Intensive Care Unit (ICU) patients stratified according to the development of an ICU-acquired pneumonia (case) or not (control) at baseline and the time of the event.

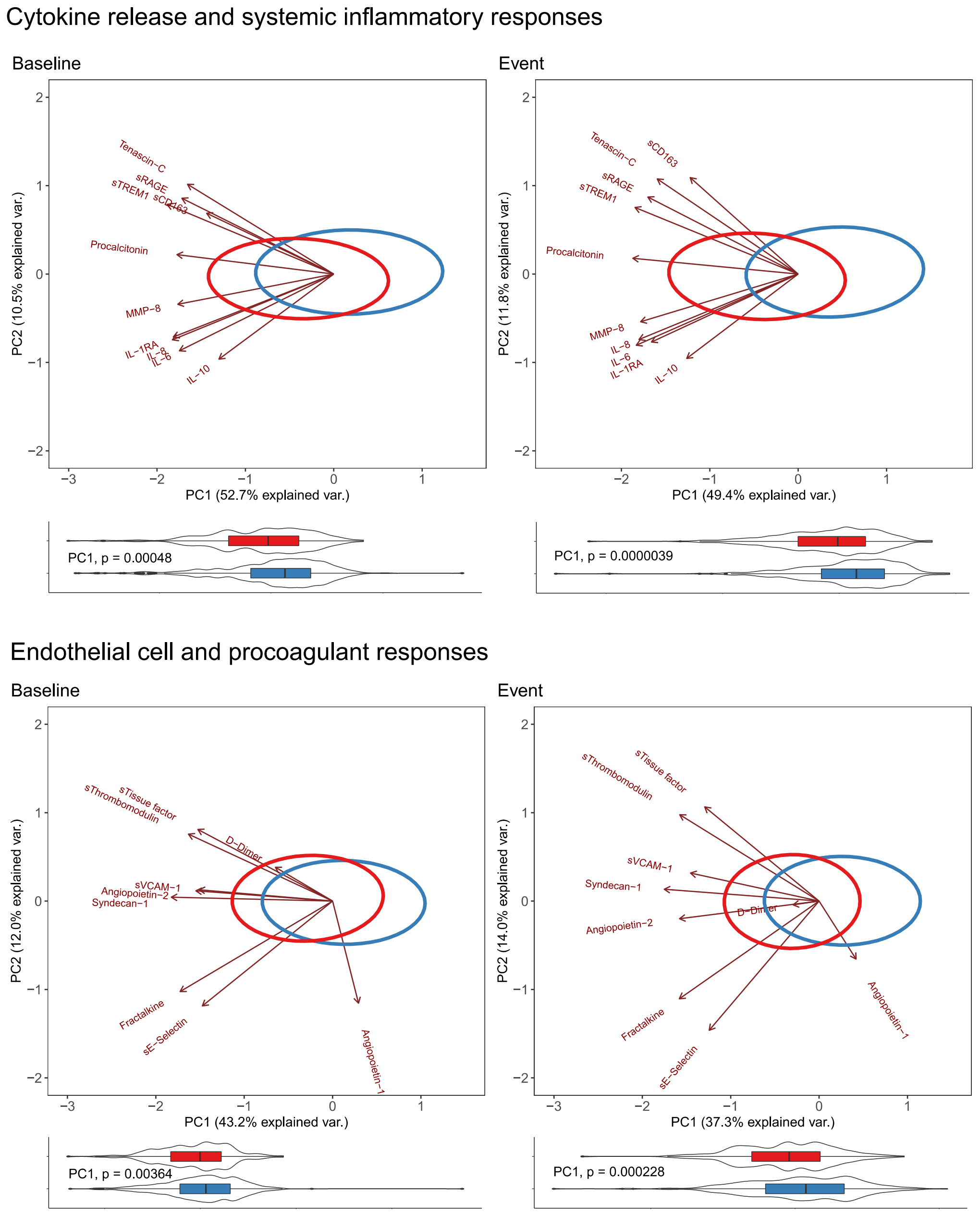

The baseline sample was obtained upon enrolment into the study. In cases, a follow up blood sample was obtained on the day the pneumonia was diagnosed (event); in controls, a follow up sample ("event") was drawn on day 7 after enrolment into the study. Data are presented as principal component analysis (PCA) plots. Ellipse circles of cases and controls in PCA plots are drawn around patient data points (not shown here for clarity), wherein the centroid is the barycenter of the patient data points belonging to the same group; arrows in PCA plots indicate positive or negative correlation of plasma markers with loadings of PCA components. The boxplots show the difference in the first component (PC1) of the PCA plot which largely and significantly explains the variance between groups (with p-values indicating group difference on PC1 calculated with Mann-Whitney-U).

Fig. S3. Cytokine release and systemic inflammatory responses in Intensive Care Unit (ICU) patients stratified according to the development of ICU-acquired pneumonia (cases, red) or not (controls, blue) at baseline and event.

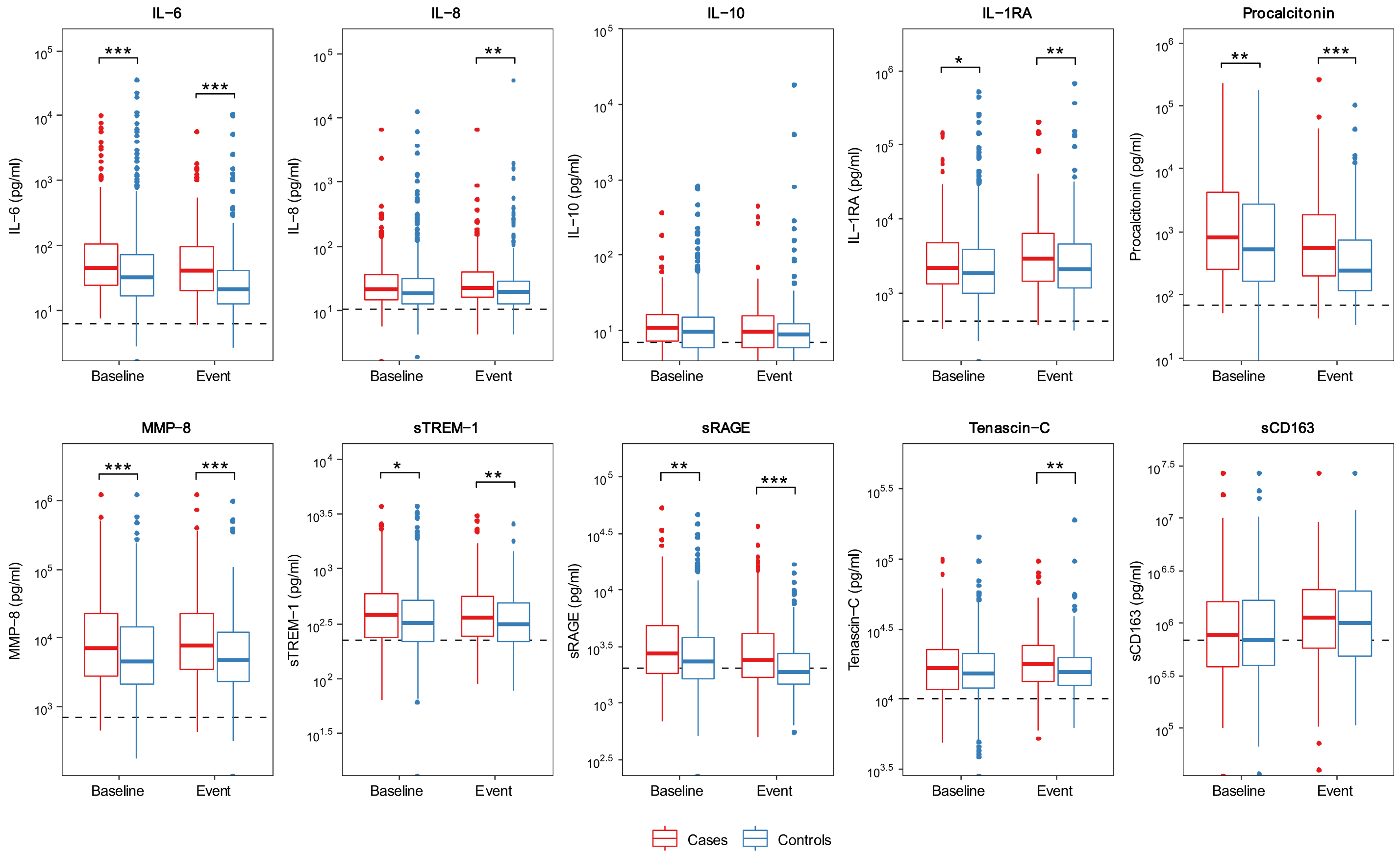

The baseline sample was obtained upon enrolment into the study. In cases, a follow up blood sample was obtained on the day the pneumonia was diagnosed (event); in controls, a follow up sample ("event") was drawn on day 7 after enrolment into the study. Data are expressed as box-and-whisker diagrams depicting the median and lower quartile, upper quartile, and their respective 1.5 interquartile range as whiskers. Dashed lines indicate median values obtained in 19 age and sex matched subjects. *P* values are derived from the mixed model taking the group, time, and their interaction as fixed effects and patients-specific intercept as random effect. Asterisks indicate differences between groups (**P* <0.05, ***P* <0.01, ****P* <0.001). *Definition of abbreviations:* CD = cluster of differentiation; IL = interleukin; MMP = matrix metalloproteinase; RA = receptor antagonist; RAGE = receptor for advanced glycation endproducts; s = soluble; TREM = triggering receptor expressed on myeloid cells.

Fig. S4. Endothelial cell and procoagulant responses in Intensive Care Unit (ICU) patients stratified according to the development of ICU-acquired pneumonia (cases, red) or not (controls, blue) at baseline and event.

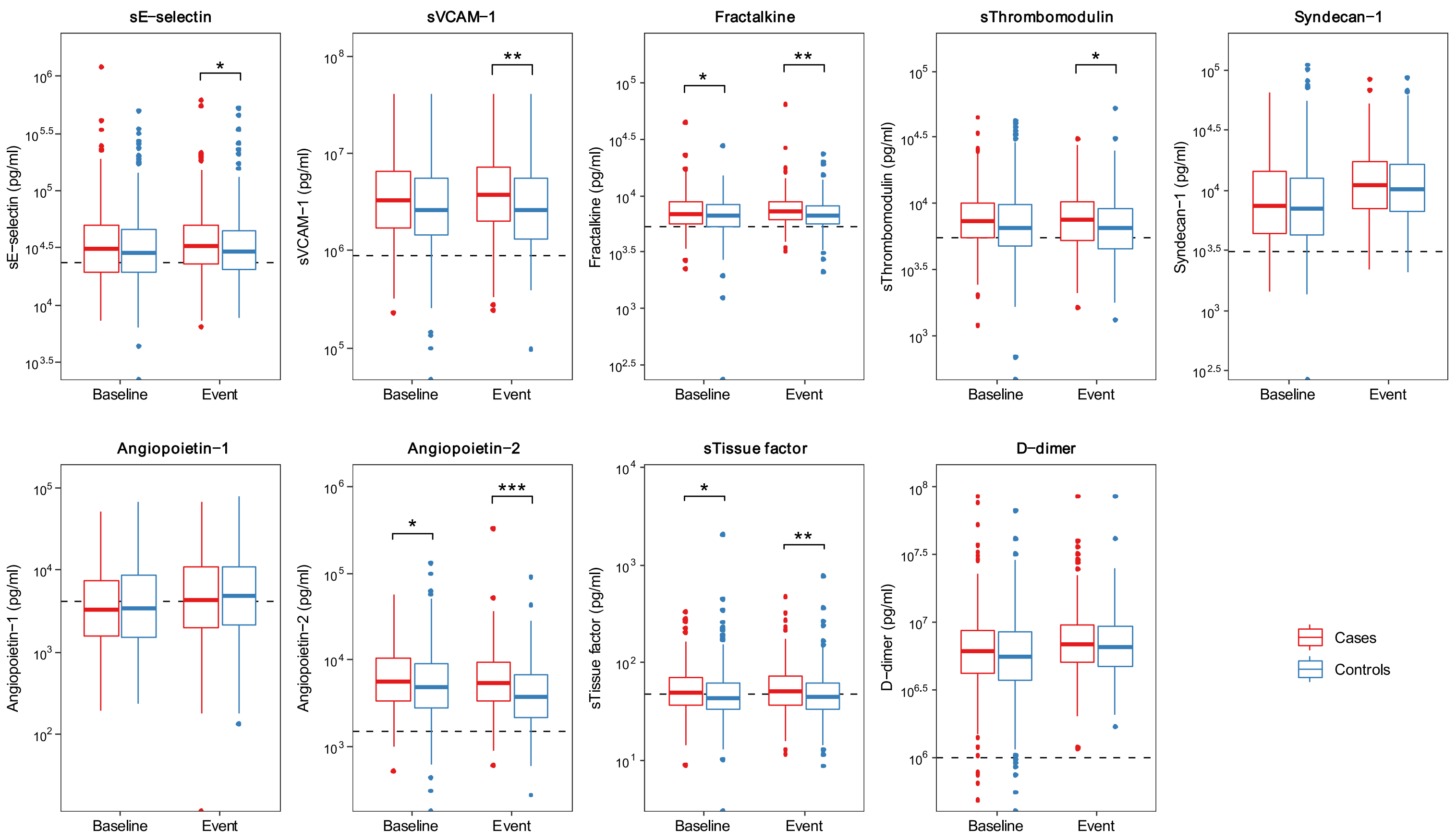

The baseline sample was obtained upon enrolment into the study. In cases, a follow up blood sample was obtained on the day the pneumonia was diagnosed (event); in controls, a follow up sample (“event”) was drawn on day 7 after enrolment into the study. Data are expressed as box-and-whisker diagrams depicting the median and lower quartile, upper quartile, and their respective 1.5 interquartile range as whiskers. Dashed lines indicate median values obtained in 19 age and sex matched subjects. *P* values are derived from the mixed model taking the group, time, and their interaction as fixed effects and patients-specific intercept as random effect. Asterisks indicate differences between groups (**P* <0.05, ***P* <0.01, ****P* <0.001). *Definition of abbreviations:* s = soluble; VCAM = vascular cell adhesion protein.

Fig. S5. Cytokine and systemic inflammatory responses in patients in whom pneumonia occurred prior to day 7 (i.e., in whom a baseline, event and post-pneumonia sample was obtained)

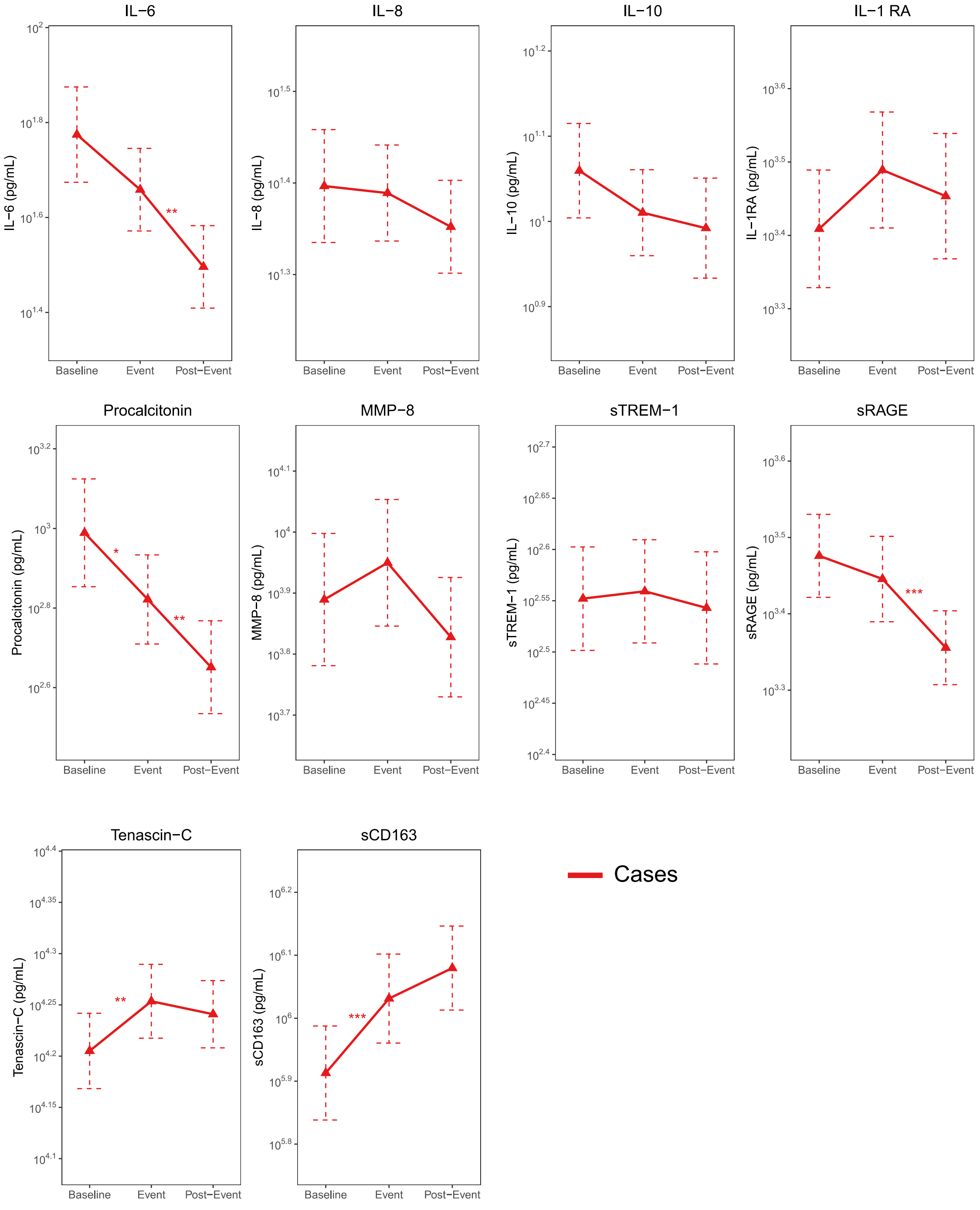

Only cases were included with paired data (baseline plus event) who also had a post-event measurement (n = 122 patients). Asterisks indicate differences between timepoints (*P <0.05, **P <0.01, ***P <0.001). *Definition of abbreviations:* CD = cluster of differentiation; IL = interleukin; MMP = matrix metalloproteinase; RA = receptor antagonist; RAGE = receptor for advanced glycation endproducts; s = soluble; TREM = triggering receptor expressed on myeloid cells.

Fig. S6. Endothelial cell and procoagulant responses in Intensive Care Unit (ICU) patients in whom pneumonia occurred prior to day 7 (i.e., in whom a baseline, event and post-pneumonia sample was obtained)

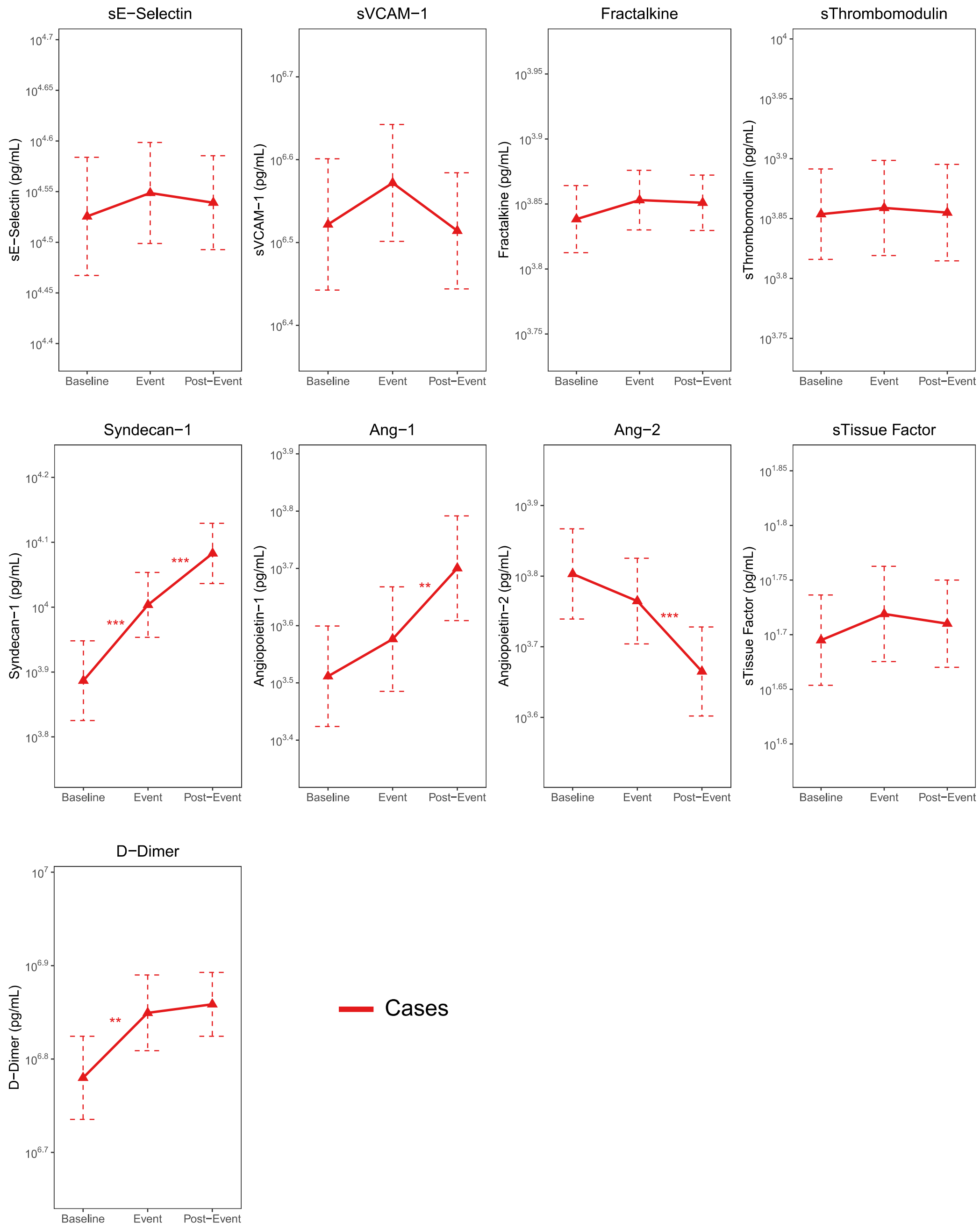

Only cases were included with paired data (baseline plus event) who also had a post-event measurement (n = 122 patients). Asterisks indicate differences between timepoints (**P <0.01, ***P <0.001). *Definition of abbreviations:* s = soluble; VCAM = vascular cell adhesion protein.

Fig. S7. Baseline cytokine release and systemic inflammatory responses in Intensive Care Unit (ICU) patients who developed ICU-acquired pneumonia.

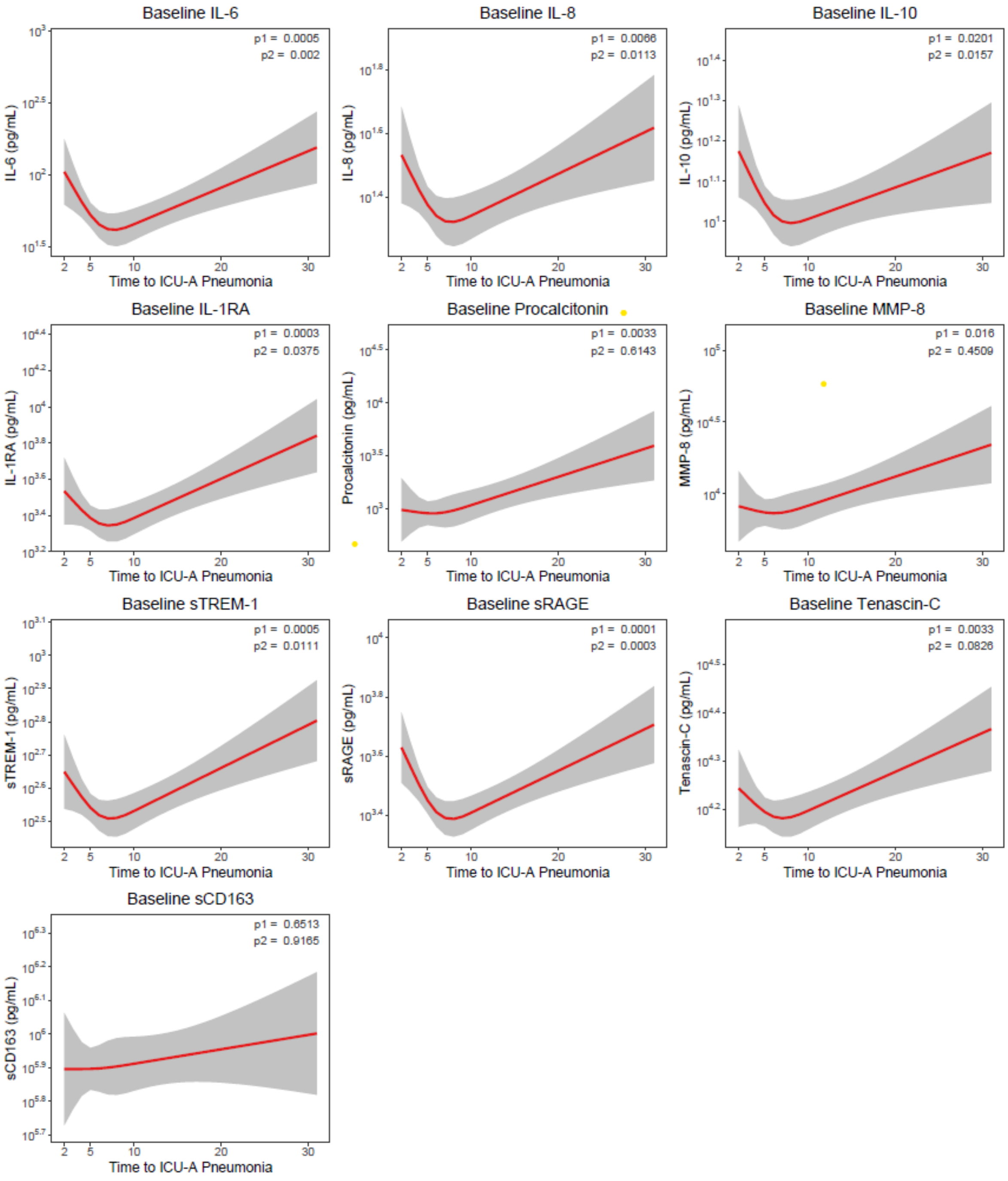

Baseline value plotted against the time of onset of the event in the ICU with 95% confidence intervals. Adjusted regression analysis for the following clinical baseline characteristics: site of enrolment, age, gender, body mass index, Charlson comorbidity index, reason for admission (medical, surgery, trauma), S. aureus colonization status, Acute Physiology And Chronic Health Evaluation (APACHE)-IV score, and immunosuppressed status. *P* value <0.05 of p1 indicates that the time of onset of pneumonia was significantly associated with the biomarker level at ICU admission. *P* value of p2 indicates nonlinearity (*P* <0.05) of the association of p1, assessed with the Wald statistic using restricted cubic splines. *Definition of abbreviations:* CD = cluster of differentiation; ICU-A = Intensive Care Unit acquired; IL = interleukin; MMP = matrix metalloproteinase; RA = receptor antagonist; RAGE = receptor for advanced glycation endproducts; s = soluble; TREM = triggering receptor expressed on myeloid cells.

Fig. S8. Baseline endothelial cell and procoagulant responses in Intensive Care Unit (ICU) patients who developed ICU-acquired pneumonia.

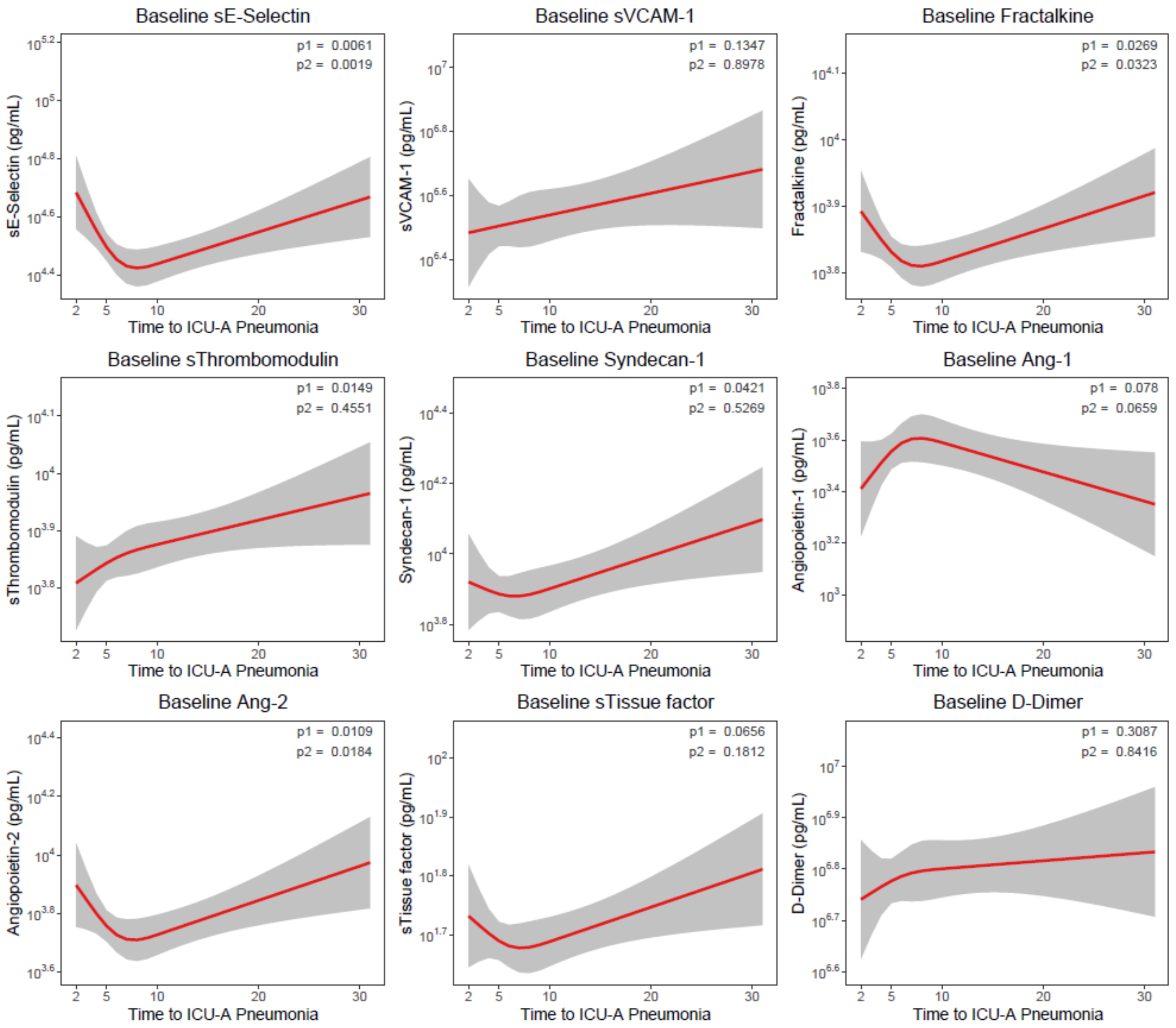

Baseline value plotted against the time of onset of the event in the ICU with 95% confidence intervals. Adjusted regression analysis for the following clinical baseline characteristics: site of enrolment, age, gender, body mass index, Charlson comorbidity index, reason for admission (medical, surgery, trauma), S. aureus colonization status, Acute Physiology And Chronic Health Evaluation (APACHE)-IV score, and immunosuppressed status. *P* value <0.05 of p1 indicates that the time of onset of pneumonia was significantly associated with the biomarker level at ICU admission. *P* value of p2 indicates nonlinearity (*P* <0.05) of the association of p1, assessed with the Wald statistic using restricted cubic splines. *Definition of abbreviations:* ICU-A = Intensive Care Unit acquired; s = soluble; VCAM = vascular cell adhesion protein.

ASPIRE-ICU STUDY TEAM

The ASPIRE-ICU study team includes Martin Wolkewitz, PhD (Institute of Medical Biometry and Statistics, University Medical Center Freiburg, Freiburg, Germany), Omar Ali, PhD (Microbial Sciences, R&D BioPharmaceuticals, AstraZeneca, Gaithersburg, Maryland), Alexey Ruzin, PhD (Microbial Sciences, R&D BioPharmaceuticals, AstraZeneca, Gaithersburg, Maryland), Leen Timbermont, PhD (University of Antwerp, Antwerp, Belgium), Christine Lammens, BSc (University of Antwerp, Antwerp, Belgium), Sebastiaan Hullegie, MD, PhD (Julius Center for Health Sciences and Primary Care, University Medical Center Utrecht, Utrecht University, Utrecht, Netherlands), Darren Troeman, MD (Julius Center for Health Sciences and Primary Care, University Medical Center Utrecht, Utrecht University, Utrecht, Netherlands), Denise van Hout, MD (Julius Center for Health Sciences and Primary Care, University Medical Center Utrecht, Utrecht University, Utrecht, Netherlands), Daniël Prins, MSc (Julius Center for Health Sciences and Primary Care, University Medical Center Utrecht, Utrecht University, Utrecht, Netherlands), Rubana Kalyani, MSc (Microbial Sciences, R&D BioPharmaceuticals, AstraZeneca, Gaithersburg, Maryland), Mark Eickhoff, MSc (Microbial Sciences, R&D BioPharmaceuticals, AstraZeneca, Gaithersburg, Maryland), Kathryn Shoemaker, MSc (Microbial Sciences, R&D BioPharmaceuticals, AstraZeneca, Gaithersburg, Maryland), Tuba Vilken, PhD (University of Antwerp, Antwerp, Belgium), Jelle Vlaeminck, MSc (University of Antwerp, Antwerp, Belgium), Jasmine Coppens, PhD (University of Antwerp, Antwerp, Belgium), Thomas van der Schalk, MSc (University of Antwerp, Antwerp, Belgium), Basil Britto Xavier, PhD (University of Antwerp, Antwerp, Belgium), Evelina Odisseeva, PhD (Military Medical Academy, Sofia, Bulgaria), Rossitza Vatcheva, PhD (University Hospital Queen Joanna, Sofia, Bulgaria), Michal Drab, MD, PhD (Hospital Kyjov, Kyjov, Czech Republic), Jaromir Vajter,MD, PhD (Motol University Hospital), Kadri Tamme, MD, PhD (Tartu University Hospital, Tartu, Estonia), Muriel Fartoukh, MD, PhD (Hospital Tenon Ap-Hp, Paris, France), Alain LePape,MD, PhD (Lyon Sud Hospital Center, Lyon, France), Mickael Landais, MD, PhD (Centre Hospitalier du Mans, Le Mans, France), Gaetan Plantefève, MD, PhD (Centre Hospitalier Victor Dupouy, Argenteuil, France), Evelina Tacconelli,MD, PhD (Universitätsklinikum Tübingen, Tübingen, Germany), Achim Kaasch,MD, PhD (Institut für Medizinische Mikrobiologie und Krankenhaushygiene, Dusseldorf, Germany), Róbert Jurkinya, MD, PhD (Josa Andras Hospital, Ny.regyh.za, Hungary), Iványi Zsolt, MD, PhD (Semmelweis University, Budapest, Hungary), Miranda van Rijen, MD, PhD (Amphia Hospital, Breda, the Netherlands), Olaf Cremer, MD, PhD (University Medical Center Utrecht, Utrecht, the Netherlands), Biljana Carevic, MD (Clinical Center of Serbia, Belgrade, Serbia), Jasna Jevdjić, MD, PhD (Clinical Center Kragujevac, Kragujevac, Serbia), Dolores Escudero, MD, PhD (Hospital Universitario Central de Asturias, Oviedo, Spain), Miguel Sanchez Garcia, MD, PhD (Hospital Clínico San Carlos, Madrid, Spain), Cristina Prat-Aymerich, MD, PhD (Hospital Universitario Germans Trias i Pujol, Badalona, Spain), Borja Suberviola-Cañas, MD, PhD (Hospital Universitario Marqués de Valdecilla, Santander, Spain), Angel Arenzana-Seisdedos MD, (Hospital Universitario Virgen Macarena, Sevilla, Spain), Hürrem Bodur, MD, PhD (Ankara Numune Training and Research Hospital, Ankara, Turkey), Cenk Kirakli, MD, PhD (Dr Suat Seren Chest Diseases Hospital, Izmir, Turkey), Ilkay Bozkurt, MD, PhD (University Hospital Ondokuz Mayis, Samsun, Turkey), and Sandra Long, MD, PhD (Royal Blackburn Hospital, Blackburn, United Kingdom).
